# Recombinant cathepsins B and L promote α-synuclein clearance and restore lysosomal function in human and murine models with α-synuclein pathology

**DOI:** 10.1186/s13024-025-00886-1

**Published:** 2025-08-29

**Authors:** Denise Balta, Anish Varghese, Susy Prieto Huarcaya, Alessandro Di Spiezio, André R. A. Marques, Jan Yağız Buchholz, Doğa Tabakacilar, Alice Drobny, Christian Werner, Wei Xiang, Rebecca Mächtel, Jan Philipp Dobert, Anna Fejtova, Franziska Richter, Melanie Küspert, Philipp Arnold, Paul Saftig, Friederike Zunke

**Affiliations:** 1 Department of Molecular Neurology, University Hospital Erlangen, Friedrich-Alexander University Erlangen-Nürnberg (FAU), Erlangen, Germany; 2 Neuroscience Institute, National Research Council (CNR), Padua, Italy; 3 iNOVA4Health, NOVA Medical School, NOVA University Lisbon, Lisboa, Portugal; 4 Department of Psychiatry and Psychotherapy, University Hospital Erlangen, Friedrich-Alexander University Erlangen-Nürnberg (FAU), Erlangen, Germany; 5 Institute of Biochemistry, Friedrich-Alexander University Erlangen- Nürnberg (FAU), Erlangen, Germany; 6 Department of Biotechnology and Biophysics, University of Würzburg, Biocenter, Am Hubland, Würzburg, Germany; 7 Department of Pharmacology, Toxicology and Pharmacy, University of Veterinary Medicine Hannover (TiHo), Hannover, Germany; 8 Institute of Functional and Clinical Anatomy, Friedrich-Alexander University Erlangen-Nürnberg (FAU), Erlangen, Germany; 9 Institute of Biochemistry, Christian-Albrechts-University Kiel, Kiel, Germany

**Keywords:** Cysteine cathepsins, Autophagy-lysosomal degradation, Alpha-synuclein, Parkinson disease, Synucleinopathies, Recombinant proteins

## Abstract

The autophagy-lysosomal pathway is crucial for maintaining homeostasis and survival of neurons, hence defects in this system have been associated with neurodegeneration, including Parkinson's disease (PD). The cysteine proteases cathepsin B (CTSB) and cathepsin L (CTSL) are involved in the clearance of various neurodegenerative disease-related proteins such as amyloid-$\:{\upbeta\:}$, huntingtin and the prion protein. While there are studies implicating CTSB and CTSL as mediators of α-synuclein/SNCA clearance, their exact roles remain unclear. We previously demonstrated that recombinant procathepsin D can enhance the clearance of pathological-aggregates of SNCA both in vitro and in vivo, as well as restoring autophagy function. These results prompted us to investigate the role of the two cysteine proteases CTSB and CTSL regarding SNCA degradation by dosing recombinant human procathepsin B (rHsCTSB) and procathepsin L (rHsCTSL) alone or in combination. We here demonstrate that both proteases are efficiently endocytosed by neuronal cells and transported to lysosomes, where they undergo maturation into active enzymes. Treatment with either rHsCTSB or rHsCTSL resulted in a reduction of different SNCA species, present in Triton-insoluble protein fractions as well as sensitive for various pathology- and structure-specific antibodies analyzed via Western blot, immunofluorescence and ELISA. These effects were found to be similar in all models used here: dopaminergic neurons derived from induced pluripotent stem cells (iPSC) of PD patients harboring the SNCA A53T mutation, ex vivo organotypic brain slices and primary neuronal cultures of human SNCA overexpressing Thy1 mice. Interestingly, our data so far do not indicate a synergistic effect of both cysteine cathepsins when applied together. As proof-of-concept for future therapeutic studies, intracranial injections of both recombinant enzymes reduced SNCA in brains of a transgenic mouse model (*Ctsd* knockout) harboring SNCA pathology. Moreover, treatment with recombinant CTSB and CTSL improved lysosomal/autophagy functions indicated by recovery of β-glucocerebrosidase (GCase) activity and SQSTM1 (p62) level. Further, SNCA-dependent synaptic defects as well as toxicity was reduced after treatment of neuronal cells. These findings suggest that enhancing lysosomal CTSB or CTSL effectively degrades pathology-associated SNCA, suggesting a potential therapeutic protease-based strategy for PD and other synucleinopathies.

**Supplementary Information:**

The online version contains supplementary material available at https://doi.org/10.1186/s13024-025-00886-1.

## Introduction

Parkinson's disease (PD) is one of the most common neurodegenerative disorders of late life and is characterized by motor and non-motor symptoms, as well as by the death of dopaminergic neurons in the *substantia nigra pars compacta* [1]. The neuronal loss observed in PD is characterized by cytoplasmic inclusions known as Lewy bodies and Lewy neurites, which contain abnormally folded α-synuclein (SNCA) [2]. SNCA is composed of three distinct regions: an amphipathic N-terminal region (residues 1–60), followed by a hydrophobic NAC region (Non-Aβ Component, residues 61–95) and an acidic C-terminal region (residues 96–140) [3, 4]. While most PD cases are idiopathic, various missense mutations in the *SNCA* gene, resulting in protein point mutations -such as A53T, A30P, and H50Q [5, 6]- as well as *SNCA* gene multiplications [7, 8], are linked to early-onset familial PD. Under physiological conditions, SNCA exists as soluble monomers or oligomers that are predominantly degraded by two main proteolytic pathways: the ubiquitin-proteasome and the autophagy-lysosomal systems [9, 10]. The latter is critically involved in clearing wildtype SNCA in its native state through chaperone-mediated autophagy [11, 12], and aggregated, insoluble forms via macroautophagy [9, 10, 13]. Once inside the acidic compartments, an array of proteases, in particular cathepsin proteases, are involved in SNCA degradation [14–16].

Lysosomal cysteine proteases cathepsin B (CTSB) and L (CTSL) are crucial for proteolysis of numerous aggregation-prone proteins, including SNCA [17]. Both enzymes are synthesized as a pre-proenzyme in the endoplasmic reticulum (ER) and delivered to the lysosome *via* the secretory pathway. In the lumen of the ER, the signal peptides are cleaved and the inactive procathepsin B (~ 45 kDa) and procathepsin L (~ 38 kDa) get glycosylated at specific asparagine sites [18, 19]. Within the Golgi, the mannose residues of the N-linked oligosaccharides are covalently modified, including phosphorylation. In the trans-Golgi network these residues are subsequently recognized by mannose-6-phosphate receptors for transport to the endosomes/lysosomes via clathrin-coated vesicles [20, 21]. Once in the endosomes/lysosomes, the pro-peptide is removed by autocatalysis or by cleavage mediated by other proteases, including cathepsin D (CTSD), resulting in an active single chain form of ~ 33 kDa for CTSB and of ~ 30 kDa for CTSL [17, 22]. Further processing by other cysteine proteases generate an active double chain form comprising a C-terminal heavy chain (~ 28 kDa) and an N-terminal light chain (~ 5 kDa) for CTSB and an N-terminal heavy chain (~ 23 kDa) and a C-terminal light chain (~ 7 kDa) for CTSL, which remain linked by hydrophobic interactions [17, 23].

While the cellular mechanisms underlying SNCA aggregation and PD etiology remain elusive, increasing evidence suggests dysfunction in the autophagy-lysosomal pathway to be central to PD pathogenesis. Numerous genetic association studies have identified several genes from the autophagy-lysosomal pathway in PD patients [24–27]. Moreover, mutations within the *GBA1* gene, encoding for the lysosomal enzyme β-Glucocerebrosidase (GCase), are involved in lysosomal storage disorder (LSD), and exhibits one of the highest risk loci for developing PD [28, 29], highlighting the importance of lysosomal function in neuronal cells. In recent years, the lysosomal cysteine protease B (CTSB) has emerged as a risk locus for PD [24, 26, 30, 31]. Variants within the *CTSB* locus have been found in PD patients [32, 33]. In vitro studies in neurons harboring a PD *GBA1* risk variant exhibit decreased protein levels of active CTSB [32]. Cathepsin activity was significantly reduced in midbrain dopaminergic neurons derived from induced pluripotent stem cells (iPSC) carrying an *SNCA* gene triplication or SNCA A53T point mutation [34]. Most likely this can be explained by a negative effect of pathological SNCA conformers on lysosomal enzyme transport and maturation, creating a vicious cycle of inefficient SNCA degradation [34] as shown before for GBA1 [35]. Other PD-associated gene variants such as *TMEM175*, Parkin (*PRKN*) and *LRRK2* have also been shown to cause a reduction in CTSB levels and enzymatic activity in cellular and mouse models [36–38], implying that the deficiency in CTSB lysosomal function is a contributing factor to PD pathology. Although it has been suggested that CTSB is involved in the lysosomal degradation of SNCA [34], its exact role is yet to be fully elucidated. It was initially proposed that CTSB activity is sufficient for SNCA degradation [15], but a later study from the same group attributed SNCA C-terminal truncations to incomplete clearance function in vitro [39]. Another recent study underlines the importance of CTSB activity not only for the clearance of SNCA fibrils but also for lysosomal function including GBA1 activity in dopaminergic neurons [40]. In regards to CTSL, previous reports indicate its direct effect on SNCA degradation in vitro, showing efficient digestion of recombinant SNCA amyloid fibrils by purified human CTSL [15, 41]. Also in yeast, CTSL protease activity was able to efficiently degrade aggregated human SNCA [42].

Considering the mounting evidence implicating CTSB dysfunction in PD and the studies supporting CTSB and CTSL proteolytic effect on SNCA, we here investigate the mechanisms underlying the effect of SNCA turnover and examine the therapeutic potential of both proteases in preventing SNCA pathology. This study involves elevating lysosomal cysteine protease level by administration of recombinant proforms of CTSB and CTSL in complementary cellular and animal models harboring SNCA pathologies. In a previous study, we demonstrated that the application of recombinant human procathepsin D (rHsCTSD) boosts SNCA degradation and restores neuronal function in diverse preclinical synucleinopathy models [16]. We here examine whether the external application of recombinant human procathepsin B (rHsCTSB) or procathepsin L (rHsCTSL) could be an alternative or complimentary strategy to promote SNCA clearance as well as neuronal and synaptic cell homeostasis. For this, we utilize SNCA overexpressing cell lines, iPSC-derived dopaminergic neurons of PD patients harboring SNCA pathology (SNCA p.A53T), primary neurons and organotypic brain slices of Thy1-SNCA overexpressing mice [43, 44] as well as proof-of-concept in vivo applications in *Ctsd*-deficient mice exhibiting SNCA aggregation [16, 45, 46]. In all models, efficient uptake and lysosomal maturation of both enzymes resulted in a decrease of SNCA level including pathology-associated conformers by treatment with the recombinant enzymes individually as well as in combination. Taken together, our data confirm that externally administered cysteine proteases CTSB and CTSL promote the clearance of SNCA species in vitro, ex vivo as well as in vivo, providing a new therapeutic approach for the treatment of synucleinopathies.

## Materials and methods

### Cathepsin B and cathepsin L cloning

HEK293-EBNA cells (Invitrogen, R620-07) were transfected with a pCEP-Pu containing proCTSB or proCTSL as described previously [16, 47] utilizing polyethyleneimine (PEI; Polysciences, 24765) in a 1:3 DNA to PEI ratio. After 48 h, expressing cells were selected with 0.25 mg/mL G-418 (Thermo Fisher Scientific, 11881-023) and 1 µg/µL puromycin for 3 weeks. Further, a high-expressing clone was selected by serial dilution. Cells were maintained in Dulbecco’s modified Eagle medium (DMEM; Life Technologies, 41965) containing 4 mM L-glutamine and 4.5 g/L glucose and supplemented with 10% fetal calf serum (FCS), 1% PenStrep (Sigma, P0781), 0.25 mg/mL G-418 and 1 µg/µL puromycin in a humidified 5% CO_2_ atmosphere at 37 °C.

### Recombinant CTSB and CTSL production and purification

Human recombinant proCTSB and proCTSL were produced as described previously [16, 47]. In brief, 4 × 10^6^ HEK293-EBNA cells stably overexpressing proCTSB or proCTSL were seeded in five 175 cm^2^ flasks with 35 mL of DMEM containing 4 mM L-glutamine and 4.5 g/L glucose and supplemented with 10% fetal calf serum (FCS), 1% PenStrep, 0.25 mg/mL G-418 and 1 µg/µL puromycin. At a cell density of 80%, the medium was replaced by 100 mL of DMEM supplemented with 2.5% FCS and 1% PenStrep per flask. One week after, the medium was harvested and vacuum-filtered (0.22 μm; Fisher Scientific, FB12566510). An Amicon system and ultracentrifugation disk (10MWCO; Millipore, PLGC07610) were used to concentrate the medium to a final volume of 50 mL. The recombinant enzymes were purified by binding its N-terminal His-tag to a HisTrap 1 mL column (GE Healthcare, 29-0510-21) on an ÄKTA pure™ 25 protein purification system (Cytiva) followed by elution with 250 mM imidazole (Millipore, 104716) in phosphate-buffered saline (PBS: 137 mM NaCl, 2.7 mM KCl, 0.8 mM Na_2_HPO_4_, 1.5 mM KH_2_PO_4_, pH 7.4) or via gravity-flow using Ni-NTA agarose beads (Macherey-Nagel, 745400.100). Size-exclusion chromatography was performed as a second purification step via a Superdex 75 column (GE Healthcare, GE17-5174-01). Enzyme purity was checked by SDS-PAGE, and the fractions containing monomeric rHsCTSB or rHsCTSL were pooled and concentrated using a Vivaspin 20 (10MWCO; Sartorious, VS2002).

### Production of recombinant SNCA

Human SNCA was expressed in in E. coli BL21 (DE3) pLysS competent cells (Novagen) using a human α-syn PT7-7 construct (a gift from Dr. Hilal Lashuel, Addgene plasmid #36046; RRID: Addgene_3604636). Purification of SNCA was performed as previously described [48]. Monomeric SNCA was purified using a SuperdexTM 75 10/300 size exclusion column and an Äkta pure™ protein purification system (Cytiva) in a running buffer of 50 mM Tris/HCl, pH 7.4 with 150 mM NaCl.

### H4 cell culture

Human H4 neuroglioma cells with tetracycline-inducible overexpression of wild-type SNCA (tet-off system) were previously characterized by Mazzulli et al. [35]. Cells were cultured in OptiMEM medium (Thermo Fisher Scientific, 31985070) supplemented with 5% tetracycline-free FCS (PAN-Biotech, P30-3602), 200 µg/mL Geneticin (Thermo Fisher Scientific, 10121035), 200 µg/mL Hygromycin (Thermo Fisher Scientific, 10687010), and 1% penicillin/streptomycin. For experiments, H4 cells were plated in 6-well plates at a density of 2 × 10⁵ cells per well for Western blot analysis or 1.2 × 10⁵ cells per well on 12-mm coverslips for immunofluorescence (IF). The following day, recombinant human CTSB (rHsCTSB), recombinant human CTSL (rHsCTSL), or a combination of both proteases (20 µg/mL) was added to the culture medium. Cells were then incubated for 24, 48, or 72 h prior to harvesting.

To inhibit lysosomal acidification, 200 nM Bafilomycin was added alongside the recombinant enzymes for the specified durations mentioned above.

### Generation of CTSB-and CTSL deficient H4 cells

CTSB or CTSL knockout cells were established by CRISPR-Cas9 following the previously published protocol by Bunk et al. [49]. Briefly, ribonucleoprotein (RNP), complexes of CTSB or CTSL multi RNA guides and Cas9 protein were assembled as per the manufacturer’s instructions (Gene Knockout Kit v2; Synthego) to transfect H4 cells via the Neon Transfection System. The multi RNA guides used in this study target exon 3 of the CTSB gene and exon 5 of CTSL gene. **CTSB**: Guide RNA (gRNA) 1: 5’ -C*C*C*ACAGCCUACCUGCCACG- 3’, gRNA 2: 5’ – A*U*G*GAAAGAGGGCCUGCUCC- 3’, gRNA 3: 5’ -U*U*C*CAACAUGUGGCAGCUCU- 3’. **CTSL**: gRNA 1: 1: 5’ -A*G*A*UAAGCCUCCCAGUUUUC- 3’, gRNA 2: 5’ – A*G*G*CUGCAAUGGUGGCCUAA- 3’, gRNA 3: 5’ -U*U*C*UGUUGCCUCAUAUGGAU- 3’ Single CTSB or CTSL KO cells were grown and expanded for the verification of successful editing efficiency by Western blot analysis and Sanger sequencing of both, CTSB exon 3 and CTSL exon 5.

Clone 7 was used for further analyses in this study. Cells were maintained as described in the section “H4 cell culture”.

### iPSC culture and neuronal differentiation

Human induced pluripotent stem cells (iPSCs) derived from Parkinson’s disease (PD) patients expressing the SNCA A53T mutation and the matched isogenic corrected line were kindly provided by Dr. R. Jaenisch (Whitehead Institute, MIT) and thoroughly characterized in previous work by Soldner et al. [50]. iPSCs were cultured on Matrigel-coated plates (Corning, 354234) using mTeSR1 (Stemcell, 85850) or mTeSR Plus medium (Stemcell, 100–0276) and differentiated into dopaminergic (DA) neurons following a well-established protocol [51]. Between days 25 and 30 of differentiation, neurons were plated onto 24-well plates coated with poly-D-lysine (Merck, P1149) and laminin (Merck, 11243217001) at a density of 3 × 10⁵ cells per well for immunofluorescence or 4 × 10⁵ cells per well on 12-mm coverslips for Western blot analysis. Neurons were maintained in Neurobasal medium (Thermo Fisher Scientific, 21103049) supplemented with NeuroCult SM1 Neuronal Supplement (Stemcell, 05711) and 1% penicillin-streptomycin until day 90. At day 60, neurons were treated with 10 µg/mL of recombinant human CTSB (rHsCTSB) or CTSL (rHsCTSL), with treatments replenished every other day in conjunction with media replacement over a period of 21–25 days.

### Animals

#### Ctsd knockout animals

Animal handling and care were performed in agreement with the German animal welfare law. Animal experiments were approved by the Ministry of Energy, Agriculture, the Environment and Rural Areas Schleswig-Holstein under the reference number V242–40,536/2016 (81–6/16). *Ctsd* knockout animals *(Ctsd* KO; *Ctsd*^*−/−*^) mice were bred from heterozygous matings and genotyped as previously described [52].

Animals were housed in individually ventilated cages under a 12-hour light/dark cycle with unrestricted access to food and water. The housing conditions were maintained at a temperature of 19–22 °C with 45–60% relative humidity. Genotyping of mice was performed on postnatal day 0 (P0). CTSD wild-type (*Ctsd* WT; *Ctsd*+/+) and CTSD knockout (*Ctsd*-/-) mice were selected for intracranial injections. Two doses were administered: the first at P1 and the second at P19. Each injection involved 10 µL of either PBS, recombinant human CTSB (rHsCTSB), or recombinant human CTSL (rHsCTSL), containing 100 µg of enzyme, delivered using a 30G micro-syringe equipped with a spacing device for a precise injection depth of 1.15 mm over 3 min.

At P1, the injection was targeted to the caudal putamen of the right hemisphere, while the P19 injection was directed to the left hemisphere. All mice were sacrificed at P23, which was chosen as the humane endpoint for untreated *Ctsd* KO mice. Following sacrifice, brains were collected, with the left hemisphere reserved for Western blot analysis and the right hemisphere used for immunofluorescence (IF).

For IF, brains were fixed in 4% paraformaldehyde (PFA) for 4 h at rt, then washed in PBS overnight at 4 °C. Subsequently, brains were immersed in a 30% sucrose solution prepared in PBS. Sagittal sections of 35 μm thickness were obtained using a Leica SM 2000 R sliding microtome (Leica Microsystems) with dry-ice cooling. Sections were stored in PBS containing 0.02% (w/v) sodium azide until further analysis.

### Thy1-SNCA animals

Animal breeding, husbandry and use of the Thy1-mice were reviewed and approved by the Government of Lower Franconia, Bavaria, Germany (RUF-55.2.2-2532-2-1489).

The breeding involved heterozygous females with wildtype males of the B6D2F1 line from Charles River on a hybrid background (C57BL/6 x DBA/21F).

All animals were housed in individually ventilated cages under a 12-hour light/12-hour dark cycle, with ad libitum access to food and water. Mice were genotyped at postnatal day 0 or 1 (for primary neuronal culture) or 10 (for organotypic brain slices).

### Generation of organotypic brain slices

Organotypic brain slices were prepared from postnatal day 10 old Thy1 mice (female and male animals).

*Cortical slice cultures*: For organotypic slice culture, 300 μm thick coronal slices were generated from forebrains of 10 days old Thy1 transgenic (tg) mice at the level of the body of the corpus callosum using a Leica VT1000S vibratome. Slices were placed on 0.4 μm Millicell-CM™ organotypic cell culture inserts (Merck-Millipore, PICM0RG50) in a 6-well plate and incubated in 1 mL culture medium containing 50% MEM (Gibco, 32360-026), 25% EBSS (Sigma, E6267), 25% heat-inactivated horse serum (Gibco, 26050-088), 1x Penicillin/Streptomycin (Anprotec, AC-AB-0024), 1x GlutaMAX (Gibco, 35050-061), 25 mM HEPES (Gibco, 15630-080), and 6.5 mg/mL D-glucose (Sigma, G8769). The medium was replaced every other day for 7 days. After 7 days of culturing, organotypic brain slices were transferred into culture medium supplemented with either 20 µg/mL rHsCTSB or rHsCTSL, including medium changes every second day for another 14 days. At day 21, the slices were fixed in 4% PFA for 20 min at room temperature (rt) and stored in PBS at 4 °C prior to IF staining.

### Primary neuronal culture

Primary cortical neurons were cultured from postnatal day 0 or 1 old Thy1 mice (female and male animals). Cortices from Thy1 mouse brains were dissected and placed in cold HBSS (ThermoFisher, 14175095). After removing HBSS, cortices were washed with fresh HBSS and then incubated in Papain-Protease-DNase solution (PPD mix), containing HBSS, 0.01% Papain (Worthington, LK003176), 0.1% Dispase II (Roche, 04942078001), 0.01% DNAse I (Worthington, LS0002139), and 12.4 mM MgSO_4_ at 37 °C and triturated thrice, at 10 min intervals. The resultant cell suspension was then passed through a 70 μm cell strainer and centrifuged at 300 x g for 3 min at rt. The resultant cell pellet was resuspended in ‘NBA media’ consisting of Neurobasal A medium (Gibco,12349-015), 0.2 mM Glutamax (Gibco,35050-038), 2% B27 (Gibco,17504-044), 0.1 M NaPyr (Gibco,11360-070), and 0.1 M Anti/Anti (Gibco, 15240-062), and centrifuged again (500 x g, 5 min, rt) twice more, with supernatant removal each time. After the final wash, cells were resuspended in 1 mL NBA mix and seeded onto 24-well plates containing coverslips pre-coated with poly-D-Lysine, at a density of 2 × 10^5^ cells per well. After 7 days in vitro (DIV7), half media changes were performed by removing half the volume of conditioned media and replenishing with fresh, pre-warmed NBA mix media. Primary neurons were treated with 20 µg/mL rHsCTSB or rHsCTSL on DIV10 and DIV12. Media was collected for performing LDH assay both prior to treatment at DIV 9 and after treatment DIV 14, respectively. Cells were harvested for ELISA and activity assays and fixed for IF analysis at DIV 15.

### LDH cytotoxicity assay

The release of lactate dehydrogenase (LDH) into the cell culture media is an indicator for damaged cells. To determine possible cytotoxic effects of rHsCTSB or rHsCTSL treated cells, the Pierce LDH Cytotoxicity Assay Kit (Thermo Fisher Scientific, 88953) was used. In short, 50 µL of sample media were collected from rHsCTSB or rHsCTSL-treated cells, transferred to a 96-well flat bottom plate (Greiner, 655083) in triplicate wells and mixed with 50 µL of reaction mixture. Following incubation for 30 min at rt, 50 µL of stop solution was added into each well. The positive control provided by the kit was used. LDH activity was measured with a CLARIOstar microplate reader (BMG Labtech) at the absorption wavelengths 490 nm and 680 nm.

### Live-cell lysosomal CTSB, CTSL, and GCase activity assay

H4 cells were seeded on a dark 96-well plate with clear bottom (Corning, 3603) at a cell density of 5 × 10^4^ cells per well. The next day, cells were treated with 20 µg/mL of rHsCTSB or rHsCTSBL for 24, 48 and 72 h. The cells were then treated with 200 nM Bafilomycin A1 (BafA1) or DMSO for 1 h at 37 °C. After washing with media, substrates for CTSB (Biorad; #ICT938), CTSL (BioRad; #ICT942), or GCase (PFB-FDGluc; Thermo Fisher Scientific, P11947) [34, 53] was incubated for 1 h at 37 °C. Afterwards, cell culture media was replaced by phenol red-free Opti-MEM (GIBCO, #11058-021) and the fluorescence intensity was measured every 30 min for 3 h by a CLARIOstar microplate reader (BMG Labtech), at excitation of 592 nm and emission of 628 nm (for CTSB and CTSL) and at 485 nm and emission of 530 nm (for GCase). After the final reading, cells were fixed in 4% PFA for 20 min, permeabilized with 0.3% Triton X-100 for 30 min, and blocked in Intercept TBS blocking buffer (Li-Cor, 927–60001) for 1 h at rt. Next, Celltag700 (Li-Cor, 926–41090) was diluted 1:500 in Intercept TBS blocking buffer with 0.1% Tween 20 and added to the cells for 1 h at rt. The plate was then washed three times with PBS and scanned using an Odyssey imaging system (Li-Cor Biosciences, Lincoln, NE, USA). For quantification, ImageStudioLite 5.2 (Li-Cor Biosciences, Lincoln, NE, USA) was used. Analysis was carried out as follows: the fluorescence intensity of the enzymatic substrate was normalized to the Cell number (CellTag700) and graphed as fluorescence intensity (y-axis) and time (x-axis). Total CTSB, CTSL, or GCase activity was calculated by the area under the curve (AUC) of DMSO and the lysosomal activity was determined by subtracting AUC of DMSO vs. BafA1. The results of the lysosomal enzyme activity measurement are depicted as bar graphs ± SEM.

### Lysate CTSB, CTSL and GCase activity assays

To measure CTSB or CTSL activity, cell pellets were freshly lysed in Triton X-100-based acidic buffer (50 mM sodium acetate, 0.1 M NaCl, 1mM EDTA, 0.2% Triton X-100, pH 4.5). 5 µg of cell lysate was incubated in 100 µL Triton X-100-based acidic buffer containing 20 µM quenched fluorogenic peptide (Enzo, #BML-P139-0010) for CTSB and 9.4 µM (BioRad; #ICT942) of fluorescent probe for CTSL for 30 min at 37 °C. To measure GCase activity, cell pellets were lysed in Triton-based buffer − 1% Triton X-100, 10% glycerol, 150 mM NaCl, 25 mM HEPES pH 7.4, 1 mM EDTA, 1.5 mM MgCl_2_ containing 1 mM PMSF, 2 mM NaVO_3_, 50 mM NaF and 1X protease inhibitor cocktail (PIC), and 1X phosphostop (Roche Diagnostics GmbH, 4906837001). 5 µg of cell lysate was incubated in 100 µL of GCase activity buffer − 0.15 M phosphate/citrate buffer, 0.25% sodium taurocholate (Sigma-Aldrich, St. Louis, MO, United States, #T9034), 0.25% v/v Triton X‐100 – and 1 µM of the GCase substrate 4‐methylumbelliferyl‐*β*‐D‐glucopyranoside (4MU, Merck Millipore, Billerica, MA, United States, #M3633). After incubation for 30 min at 37 °C, the reaction was stopped using 100 µL stop solution (0.1 M glycine, pH 10.4). Enzyme activities were measured using SpectraMax Gemini (Molecular Devices, San José, CA, United States) or ClarioStar (BMG LABTECH, Ortenberg, Germany) plate-readers at at excitation: 365 nm and emission: 440 nm for CTSB, excitation: 590 nm and emission: 628 nm for CTSL, and excitation: 365 nm and emission: 445 nm for GCase. All values were corrected for background fluorescence.

### Sequential protein extraction protocols

1. Our primary solubility assay, generating Triton soluble (T-sol) and Triton-insoluble (T-insol)/ SDS-sol fractions, was performed as previously described [54]. H4, iPS-derived dopaminergic neurons (DA-iPSn), or frozen whole brain tissue were lysed in a Triton-based buffer (1% Triton-X100, 10% glycerol, 150 mM NaCl, 25 mM HEPES pH 7.4, 1 mM EDTA, 1.5 mM MgCl_2_) containing 1 mM PMSF, 2 mM NaVO_3_, 50 mM NaF and 1X protease inhibitor cocktail (PIC), and 1X phosphostop (Roche Diagnostics GmbH, 4906837001) by incubation on ice-water slurry for 30 min to obtain the T-sol fraction from the supernatant. Following three cycles of freezing (-80°C) and thawing (37°C), the samples were ultracentrifuged at 100,000 x g, 4 °C for 30 min. T-insol/ SDS-sol fraction was generated by lysing the residual pellet in 50 mM Tris, pH 7.4 buffer containing 2% SDS and 1X PIC by boiling at 99 °C for 10 min, sonicating three times and ultracentrifuged at 100,000 x g, 22 °C for 30 min. Protein concentrations of the T-sol and T-insol fractions were determined using a BCA assay kit (Thermo Fisher Scientific, 23225) according to manufacturer’s instructions.
2. Additionally, an alternative sequential extraction protocol based on solubility of SNCA in RIPA buffer and urea/SDS was performed as described previously [55]. Pellets from iPS-derived dopaminergic neurons (DA-iPSn) were homogenized in TBS (50mM Tris, pH7.4, 175mM NaCl, 5mM EDTA) buffer containing 1% Triton X-100 and freshly added − 1 mM PMSF, 2 mM NaVO_3_, 50 mM NaF, 1X protease inhibitor cocktail (PIC), and 1X phosphostop (Roche Diagnostics GmbH, 4906837001), by incubation on ice water slurry for 30 min. After three cycles of freezing and thawing, the samples were ultracentrifuged at 100,000 g, 4 °C for 1 h to obtain the TBS/Triton soluble (TBS/Triton-sol) fraction. The pellet obtained was then resuspended in radioimmunoprecipitation assay (RIPA) lysis buffer (50 mM Tris/HCl pH 8.0, 150 mM NaCl, 5 mM EDTA, 1% NP-40, 0.5% sodium deoxycholate) containing 0.1% SDS, and freshly added 1X protease inhibitor cocktail (PIC), and 1X phosphostop and then ultracentrifuged at 100,000 g, 4 °C for 1 h to obtain the RIPA-soluble (RIPA-sol) fraction. The pellet obtained was then resuspended in 8 M Urea/5% SDS to obtain the urea/SDS fraction.

### Western blot analysis

T-sol and T-insol lysates (40 µg protein for H4 cells and 30–40 µg protein for DA-iPSn) were denatured with 5X Laemmli buffer were mixed with 5X Laemmli buffer (0.3 M Tris-HCl, pH 6.8, 10% w: v SDS, 50% v: v glycerol, 5% v: v ß-mercaptoethanol, 5% w: v bromophenol blue) at a ratio of 1 part 5X Laemmli buffer to 4 parts sample and boiled for 5 min at 95 °C. Samples were run by electrophoresis on 12% SDS-PAGE gels at 125 V. Subsequently, proteins were transferred onto PVDF membranes (Millipore, IPFL00010) at a constant voltage of 30 V for 1 h. The membranes were fixed in 0.4% paraformaldehyde for 20 min, blocked in Intercept blocking buffer (Li-Cor, 927-60001) for 1 h and incubated overnight with primary antibodies in the appropriate antibody diluent (Li-Cor, 927-65001). The membranes were washed with TBS-Tween 20 (0.1%) and incubated with secondary fluorescent-conjugated antibodies for 1 h at rt. Detailed information about all applied primary and secondary antibodies for western blotting can be found in Table 1. Detection of the blots was performed by using the Amersham Typhoon Biomolecular Imager (GE Lifesciences) or Odyssey (LI-COR Biosciences, Lincoln, NE, USA) imaging systems. CBB-stained SDS-PAGE gels were used to check protein loading.

**Table 1:** Details of the primary and secondary antibodies used for Western blotting

Antibody	Host species	Company	Catalogue number	Dilution
***Primary antibody***				
SNCA (C-20)	Rabbit	Santa Cruz	sc-7011-R	1:1,000
SNCA (syn-1)	Mouse	BD	610,787	1:500
SNCA (Syn-303)	Mouse	Biolegend	824,301	1:500
SNCA (Polycloncal)	Rabbit	Proteintech	10842-1-AP	1:1,000
SNCA (pSer129)	Rabbit	Cell Signaling	23,706	1:500
CTSB	Goat	R&D systems	AF953	1:500
CTSL	Goat	R&D systems	AF952	1:500
ACTB	Mouse	Sigma	A5441	1:1,000
TUBB3	Mouse	BioLegend	802,001	1:1,000
GAPDH	Goat	Cell signaling Technology	14C10	1:1,000
SQSTM1	Mouse	Abcam	ab56416	1:500
NSE	Mouse	BioLegend	804,901	1:500
***Secondary antibody***				
Alexa Fluor 680 anti-mouse	Donkey	Thermo Fisher Scientific	A-100,038	1:10,000
Alexa Fluor 680 anti goat	Donkey	Thermo Fisher Scientific	A-21,084	1:10,000
IRDye 680 anti rabbit	Donkey	Li-Cor	926-68073	1:10,000
IRDye anti mouse 800	Donkey	Li-Cor	926-32212	1:10,000
IRDye anti goat 800	Donkey	Li-Cor	926-32214	1:10,000
IRDye anti rabbbit 800	Donkey	Li-Cor	926-32213	1:10,000
Cy3^®^	Donkey	Abcam	ab6949	1:10,000

### Enzyme-linked-immunosorbent assay (ELISA)

Aggregated SNCA and total human SNCA in DA-iPSn and primary neurons from the Thy1-SNCA was detected and quantified using an SNCA aggregate ELISA kit (BioLegend, 449407) and total human SNCA kit (BioLegend, 448607) respectively, following the manufacturer’s protocol. For this, total lysate was generated by lysing the cell pellets in 50 µL of Triton-based buffer (1% Triton-X100, 10% glycerol, 150 mM NaCl, 25 mM HEPES pH 7.4, 1 mM EDTA, 1.5 mM MgCl_2_) supplemented with 1X PIC and 1X phosphostop. The lysates were incubated on an ice-water slurry for 30 min and subjected to three cycles of freezing and thawing. Subsequently, the samples were centrifuged at 12,000 x g for 15 min, and the supernatant was collected. Protein concentration was determined using a BCA assay, and 30 µg of protein from the lysates was used for the aggregated SNCA detection ELISA assay and 0.1 µg of protein was used for the total human SNCA ELISA assay. Absorbance was measured within 15 min using a CLARIOstar microplate reader (BMG Labtech) at 450 nm and 570 nm.

### *In vitro* digestion of SNCA

The preparation and characterization of SNCA fibrils from purified monomeric human recombinant SNCA were carried out as described previously [56]. Monomeric SNCA was diluted to 1 µg/µL with Tris/HCl buffer (0.1 M, pH 7.4) in a reaction tube (1.5 mL) in the presence of a 3 mm polytetrafluoroethylene (PTFE) bead (Polysciences, #17649-100). The reaction tube was shaken (Eppendorf Thermomixer Compact 5350, Hamburg, Germany) at 37 °C and 1000 rpm for 3 days. SNCA fibril generation was validated using ThioflavinT (ThioT) assay. For this, 10 µL of each sample was transferred into a 96-well plate (Black Microwell SH, Thermo Scientific #437111) and mixed with 10 µM of ThioT solution for a total volume of 100 µL in Tris/HCl buffer (0.1 M, pH 7.4). Samples were measured in triplicates using a SpectraMax Gemini EM (Molecular Devices, San Jose, USA) at 410 nm excitation and 475 nm emission.

For the digestion, 2 µg SNCA (monomers or fibrils; final concentration: 3.5 µM) were added to 30 µL Triton X-100-based buffer (50 mM sodium acetate, 0.1 M NaCl, 1mM EDTA, 0.2% Triton X-100, pH 4.5) alongside 0.44 µg of rHsCTSB (final concentration: 0.3 µM), 0.34 µg of rHsCTSL (final concentration: 0.3 µM) or a combination of rHsCTSB and rHsCTSL together (final concentration: 0.3µM for each cathepsin). The mixture was incubated at 37 °C for 0.25 h, 0.5 h, 1 h, 2 h, 4 h, 8 h, 24 h, 48–72 h before terminating the reaction by adding denaturing 1X Laemmli buffer and heating the samples to 95 °C for 5 min. As undigested controls, reaction mixtures without rHsCTSB or rHsCTSL in the Triton X-100-based buffer were prepared in the similar manner. The samples were separated on a 15% Tris-Glycine gels via SDS-PAGE and analyzed using CBB staining.

### Immunofluorescence

H4 cells or DA-iPSn, or primary neurons from Thy1-SNCA model, were fixed with 4% PFA for 20 min at rt, followed by permeabilization with 0.3% Triton X-100 in PBS for 30 min. To prevent nonspecific binding, cells were blocked for 1 h at rt in a solution containing 2% BSA and 5% FCS in PBS with Triton X-100. Primary antibodies were prepared in the blocking buffer and applied to the cells for overnight incubation at 4 °C. After washing with PBS containing Triton X-100, cells were treated with secondary antibodies diluted 1:500 in blocking buffer and incubated at rt for 1 h.

*Sagittal slices* from mouse brain were permeabilized with 0.3% Triton X-100 in PBS for 30 min, blocked in 3% BSA in PBS-Triton X-100 and incubated overnight at 4 °C with primary antibodies. Secondary antibodies were prepared at a 1:300 dilution in blocking buffer.

*Cortical brain slices* were permeabilized and blocked for 3 h at rt in solution containing 10% FCS, 1% BSA, and 0.5% Triton X-100 in PBS. Primary antibodies, diluted in the blocking buffer, were applied and incubated overnight for 4 days at 4 °C.

The following day, the slices were washed six times for 20 min each with PBS containing 0.05% Triton X-100 at rt. Secondary antibodies, diluted in blocking solution, were subsequently applied to the slices and incubated for another 4 days at 4°C. Detailed information on the primary and secondary antibodies can be found in Table 2.

Cells and brain slices were mounted either directly onto slides utilizing DAPI-Fluoromount G (SouthernBiotech, 0100 − 20) or pre-incubated with DAPI (Sigma, D8417) diluted 1:10,000 in PBS for 10 min at rt, following three washing steps with PBS. Slices were mounted with ProLong™ Gold antifade reagent. Mounted cells or slices were imaged with a confocal laser scanning microscope (IX83, Olympus *or* LSM 780, Carl Zeiss Microscopy GmbH).

ImageJ sofware was used to analyse all IF images, with uniform adjustments applied to enable direct comparisons. To analyse colocalization, H4 cells were identified within a region of interest and colocalization of two stainings (LAMP2/CTSB) was assessed using the Pearson correlation coefficient [57]. Colocalization is indicated by positive values which describe a positive correlation between both stainings.

The levels for SNCA in the different cellular models (here: LB509, pSer129, or MJFR 14-6-4-2) were assessed by quantifying the fluorescence intensity signal within a defined region of interest. Analysis of SNCA aggregates in the *Ctsd* KO mouse brain was performed semi-automatically by thresholding to a similar fluorescence intensity threshold to measure their length (µm). Each aggregate was then measured manually by drawing a straight line from top to bottom. A region of interest was subsequently delineated around each aggregate to quantify SNCA fluorescence intensity. For the mouse tissue, the analysis of MJFR 14-6-4-2-positive vesicles was conducted using a methodology similar to that employed for SNCA aggregates. Vesicle size was determined by applying a consistent fluorescence intensity threshold for quantification and length measurement. The neurite or neuronal structures containing MJFR 14-6-4-2-positive vesicles were measured for their total length. To account for structural variations, the total number of MJFR 14-6-4-2-positive vesicles was normalized to the length of the analyzed neuronal structure.

**Table 2:** Detailed information about primary and secondary antibodies utilized in Immunoflourescence

Antibodies used for human H4,DA-iPSn and primary neurons	Host species	Company	Catalogue number	Dilution
***Primary antibody***				
SNCA (LB509)	Mouse	abcam	27.766	1:100
SNCA (pSer129)	Rabbit	Cell Signaling	23,706	1:500
SNCA (syn-1)	Mouse	BD	610,787	1:500
Tyrosin hydroxylase (TH)	Rabbit	Millipore	657,012	1:100
Lamp2	Mouse	DSHB	H4B4	1:250
Neurofilament	Mouse	Biolegend	SM1 32R	1:250
CTSB	Goat	R&D systems	AF953	1:500
Synapsin-1 (SYN-1)	Rabbit	Invitrogen	A6442	1:50
TUBB3	Mouse	BioLegend	802,001	1:1,000
***Secondary antibody***				
Alexa Fluor anti mouse 594	Donkey	Invitrogen	A21203	1:500
Alexa Fluor anti rabibt 594	Donkey	Invitrogen	A21207	1:500
Alexa Fluor anti mouse 568	Donkey	Invitrogen	A10037	1:500
Alexa Fluor anti rabbit 488	Donkey	Invitrogen	A21206	1:500
Alexa Fluor anti goat 488	Donkey	Invitrogen	A11055	1:500
Alexa Fluor anti rabbit 647	Donkey	Dianova GmbH	711-605-152	1:500
Cy5^®^	Goat	Abcam	Ab6566	1:500
**Antibodies used for mouse tissue**	**Host species**	**Company**	**Catalogue number**	**Dilution**
***Primary antibody***				
SNCA filament MJFR 14-6-4-2	Rabbit	Abcam	ab209538	1:1,000
SNCA (LB509)	Mouse	abcam	27.766	1:100
Neurofilament	Mouse	Biolegend	SM1 32R	1:1,000
CTSB	Goat	R&D systems	AF953	1:500
CTSL	Goat	R&D systems	AF952	1:500
***Secondary antibody***				
Alexa Fluor anti mouse 594	Donkey	Invitrogen	A21203	1:300
Alexa Fluor anti rabbit 488	Donkey	Invitrogen	A21206	1:300
Alexa Fluor anti mouse 568	Donkey	Invitrogen	A10037	1:500
Alexa Fluor anti mouse 647	Donkey	Dianova GmbH	715-605-151	1:500
Alexa Fluor anti rabbit 568	Donkey	Invitrogen	A10042	1:500
Alexa Fluor anti rabbit 647	Donkey	Dianova GmbH	711-605-152	1:500
Alexa Fluor anti goat 488	Donkey	Invitrogen	A11055	1:500

### Structured illumination microscopy (SIM)

For the visualization of Synapsin-1 (SYN-1) and SNCA in primary neurons, SIM imaging was used. Imaging was performed using a Zeiss Elyra 7 system equipped with ZEN software and applying the SIM^2^ algorithm. Samples were visualized with a 63x/1.40 NA oil immersion Plan-Apochromat objective, using laser exctiations at 488 nm and 561 nm. Fluoresence signals were collected over 13 phase shifts and captured on a PCO Edge 4.2 M sCMOS camera passing appropriate emission filters. SIM image reconstruction was performed in ZEN Black software (Carl Zeiss Microscopy GmbH, Jena, Germany). Chromatic aberration was corrected using affine transformations derived from z-stacks of Tetraspeck beads (Invitrogen). Final adjustments to image brightness and contrast were made in FIJI, using linear scaling [58].

### Statistical analysis

All statistical analyses were performed using GraphPad Prism version 8 (Graph Pad Software, Inc., San Diego, USA). Data were analyzed by one-way analysis of variance (ANOVA) followed by Dunnett’s or Tukey’s post hoc for multiple comparisons or by two-sided, unpaired Student’s t-test for two groups comparisons as indicated in the figure legends. If not stated otherwise, data are expressed as mean ± SEM, *p* < 0.05 as considered statistically significant.

## Results

### rHsCTSB and rHsCTSL are delivered to lysosomes and functionally active in H4 cells

**Fig. 1:**
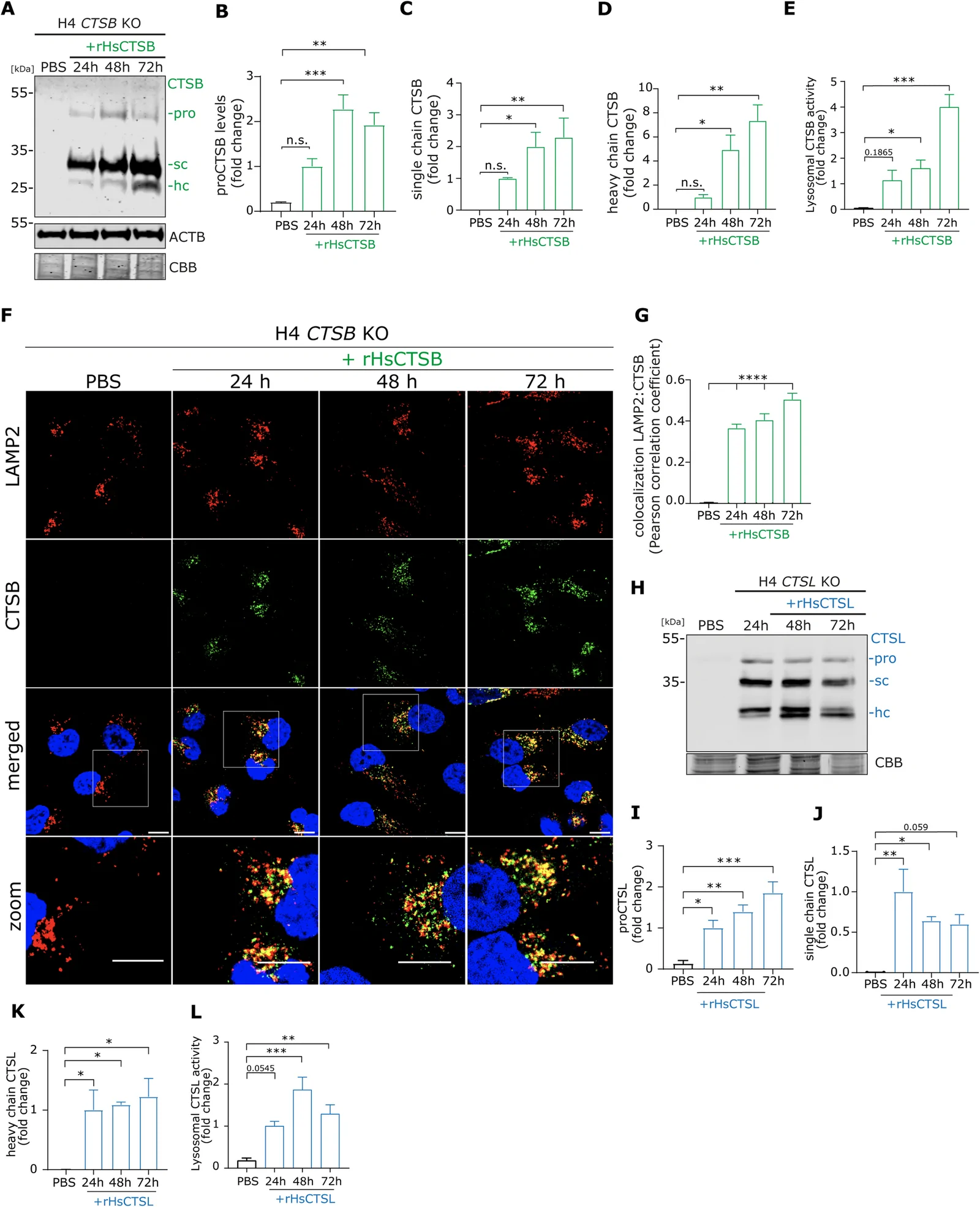
**Uptake,** **maturation** **and functional** **activity** **of** **rHsCTSB** **and** **rHsCTSL** **in H4 cells** **deficient** **for** ***CTSB*** **or** ***CTSL***. **(A)** Representative Western blot analysis of H4 *CTSB* KO cells treated with PBS or 20 µg/mL rHsCTSB for 24, 48, and 72 h. Quantification of **(B)** proform (~45 kDa), **(C) **single chain (sc, ~33 kDa), and **(D)** heavy chain (hc, ~28 kDa) of CTSB was normalized to β-actin (ACTB) and expressed as fold change relative to 24 h rHsCTSB uptake (n = 3). A schematic of CTSB maturation and protein sizes is shown in Figure S1A. Coomassie Brilliant Blue (CBB)-stained SDS-PAGE gel served as an additional loading control. **(E)** Lysosomal CTSB activity in H4 *CTSB* KO cells treated with rHsCTSB (n = 4) was measured and expressed as fold change relative to 24-hour treatment. **(F) **IF staining of LAMP2 (red) and CTSB (green) shows colocalization in PBS- and rHsCTSB-treated H4 *CTSB* KO cells. Scale bar: 10 µm. **(G)** Colocalization of LAMP2 and CTSB was quantified (n = 11–20 cells/group). **(H)** Representative Western blot analysis of H4 *CTSL* KO cells treated with PBS or 20 µg/mL rHsCTSL for 24, 48, and 72 h revealed quantification of **(I) **proform (~38 kDa), **(J) **single chain (~30 kDa), and **(K)** heavy chain (~23 kDa) of CTSL, normalized to CBB and expressed as fold change relative to 24-hour rHsCTSL uptake (n = 3). CTSL maturation and protein sizes are detailed in Figure S1F. **(L)** Lysosomal CTSL activity was measured in H4 CTSL KO cells treated with rHsCTSL (n =4) and expressed as fold change to 24-hour treatment. Data represent mean ± SEM. Statistical analysis was performed using one-way ANOVA with Dunnett’s test relative to PBS treatment. ****p < 0.0001, ***p < 0.001, **p < 0.01, *p < 0.05, n.s. = not significant

Before evaluating the therapeutic potential of the recombinant enzymes, we describe the purification as well as quality control measures of the in-house produced recombinant proforms of CTSB and CTSL. The proform was used for the treatment, as it does not exhibit proteolytic activity until reaching the acidic environment of the lysosome, preventing unwanted enzymatic activity outside the cell. Recombinant human proCTSB (rHsCTSB) was produced as described previously in Marques et al. [47]. Enzyme purity was confirmed by Coomassie Brilliant Blue (CBB)-stained SDS-PAGE gels, which showed ~ 45 kDa bands after size exclusion chromatography, corresponding to the proform of CTSB (Figure S1A, S1B). Size exclusion chromatography fractions containing CTSB were pooled and assessed for CTSB activity using a fluorogenic peptide-based assay at pH 4.5, which simulates the acidic environment of the lysosome. Turnover of fluorogenic substrate was confirmed and could be abolished by addition of Leupeptin (Figures S1C), a cysteine protease inhibitor [59]. To evaluate whether rHsCTSB is endocytosed and further processed to its active mature form, H4 cells deficient in CTSB (H4 *CTSB* KO) were treated with 20 µg/mL rHsCTSB up to 72 h. Western blot analysis revealed the presence of CTSB proform (~ 45 kDa) in whole-cell lysate within 1 h after the start of treatment from 0.5 to 72 h (Figure S1D), confirming efficient uptake of rHsCTSB. Intracellular maturation of the recombinant enzyme into the active- single chain form (~ 33 kDa) was detected after 6 h of rHsCTSB incubation (Figure S1D). The single chain form was further processed into the double chain form, with the heavy chain (~ 28 kDa) visible after 24 h on Western blot (Figure S1D, 1A). All three protein forms of CTSB were significantly increased in the cell lysate 48 and 72 h post-rHsCTSB treatment (Fig. 1A – 1D). To validate the enzymatic activity of the recombinant enzyme inside lysosomes, we performed a live-cell CTSB activity assay in H4 *CTSB* KO cells. In line with the aforementioned protein maturation results (Figure S1D, 1A), lysosomal CTSB activity was augmented after 48 h of treatment (Fig. 1E). Immunofluorescence (IF) analysis revealed a considerable colocalization between CTSB (green) and the lysosomal marker LAMP2 (red), revealing an effective delivery of rHs CTSB to the lysosome (Fig. 1F and Fig. 1G). Furthermore, a lactate dehydrogenase (LDH) assay was performed to evaluate potential cytotoxicity associated to rHsCTSB treatment. No toxicity was detected after treatment with 20 µg/mL recombinant enzyme at any time point (Figure S1E). For purification of recombinant human proCTSL (rHsCTSL) a similar approach as described for rHsCTSB was used (Figure S1F, S1G). The activity of the recombinant enzyme was evaluated using the fluorogenic peptide-based approach at pH 4.5 (Figure S1H). Uptake, maturation and lysosomal activity of rHsCTSL were assessed in H4 cells deficient for CTSL (H4 *CTSL* KO). Upon treatment with 20 µg/mL rHsCTSL, we observed the presence of CTSL proform (~ 38 kDa) in whole-cell lysate within 30 min post-treatment (Figure S1I), confirming the efficient uptake of rHsCTSL. Processing into the single chain form (~ 30 kDa) was also detected after 30 min. Further proteolytic maturation into the heavy chain (~ 23 kDa) was detectable within 2 h after treatment (Figure S1I). The levels of all three CTSL forms were significantly increased in the cell lysates 24–72 h after treatment (Fig. 1H - 1K). Lysosomal live-cell CTSL activity was also significantly elevated after 48 h (Fig. 1L). Treatment with 20 µg/mL rHsCTSL did not exert any overt cellular toxicity at any of the measured time points (Figure S1J).

Taken together, our results demonstrate that in-house produced rHsCTSB and rHsCTSL are taken up, matured, and delivered to the lysosome of H4 cells where they both exert enzymatic activity without causing cellular toxicity.

### rHsCTSB and rHsCTSL decrease SNCA in triton-insoluble/ SDS-soluble fraction in H4 cells

Previous in vitro studies suggest that both CTSB and CTSL are involved in the proteolysis of SNCA [15, 39]. To confirm these in vitro findings, we compared the degradative potential of recombinant CTSB and CTSL by incubation with recombinant SNCA conformers at pH 4.5. Generation of SNCA fibrils were confirmed using Thioflavin T fluorescence assay (Figure S2A). Consistent with previous findings, we found that rHsCTSB completely degrades monomeric SNCA efficiently with no degradation products being visible after 30 min (Figure S2B, S2C). Compared to the SNCA monomer, the degradation ability of CTSB on fibrillar SNCA was less efficient as lower molecular weight species were still present after 72 h of incubation (Figure S2B, S2D). Interestingly, rHsCTSL was found to degrade both soluble monomeric and fibrillar conformers of SNCA completely after 48 h (monomer) and 24 h (fibril) incubation time, respectively (Figure S2B, S2E, S2F). A combined treatment with both cathepsins resulted in absence of visible SNCA monomer bands after 15 min (Figure S2G), consistent with the findings for CTSB alone (Figure S2C). Additionally, no detectable SNCA fibril fragments were observed after 24 h of treatment (Figure S2H), similar to the effects seen with rHsCTSL treatment in Figure S2F.

**Fig. 2:**
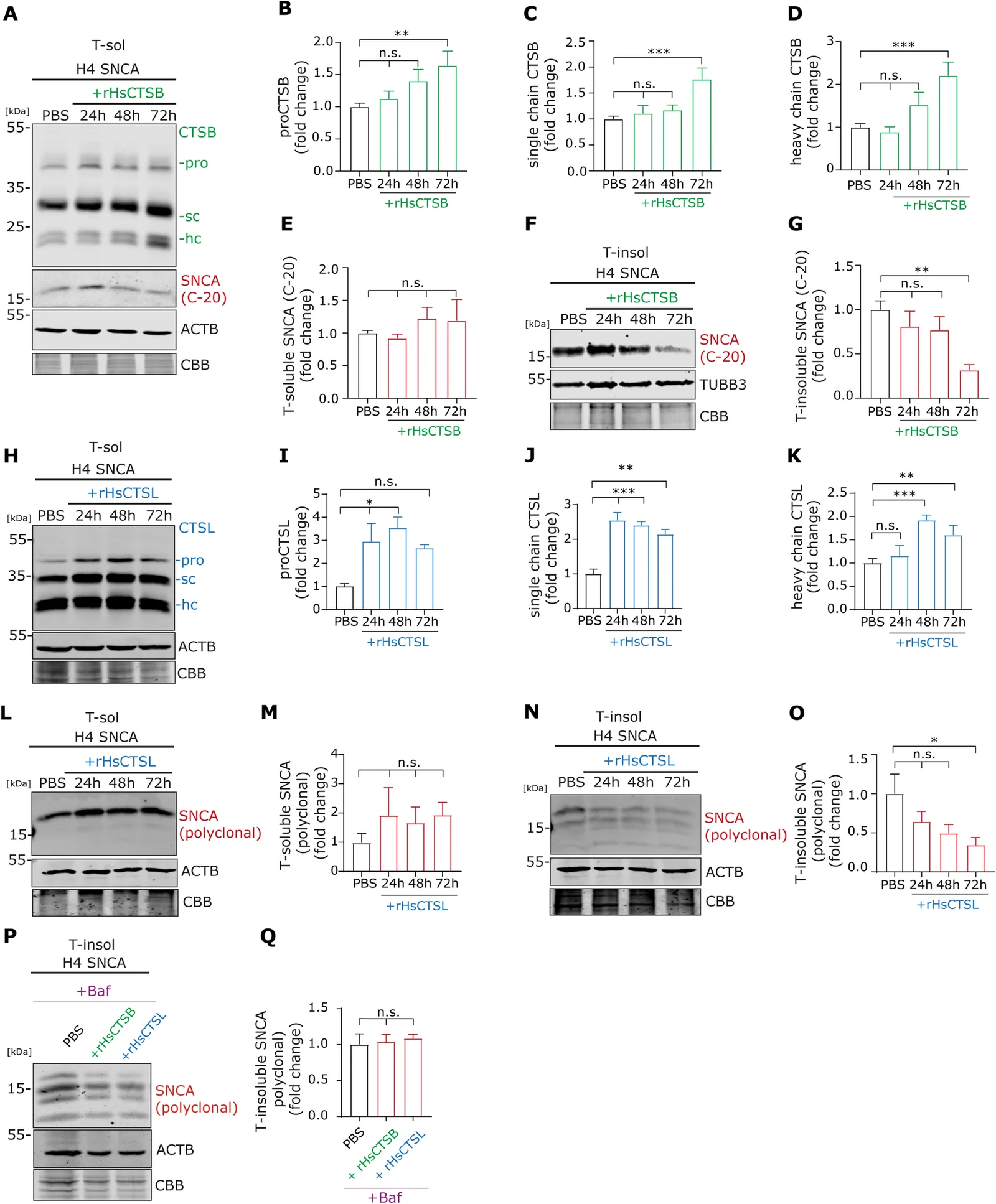
**Treatment with rHsCTSB and rHsCTSL decreases Triton-insoluble/ SDS-soluble SNCA in H4 cells overexpressing SNCA.** **(A)** Representative Western blot of Triton-soluble (T-sol) lysates from H4 cells treated with PBS or 20 µg/mL rHsCTSB for 24, 48, and 72 h, stained for CTSB and SNCA using the C-20 antibody. ACTB and Coomassie Brilliant Blue (CBB) staining served as loading controls. Quantification of **(B)** proform, **(C)** single chain (sc), **(D)** heavy chain (hc) of CTSB, and **(E)** T-sol SNCA normalized to ACTB (n = 3). **(F) **Representative Western blot of Triton-insoluble/SDS -soluble (T-insol) lysates after PBS or rHsCTSB treatment, with TUBB3 and CBB as loading controls. **(G)** Quantification of insoluble SNCA normalized to TUBB3 (n = 3). **(H)** Representative Western blots of T-sol lysates after PBS or rHsCTSL treatment for 24, 48, and 72 h, stained for CTSL. Quantification of **(I)** proform, **(J)** single chain, **(K)** heavy chain of CTSL. **(L)** Representative Western blot of T-sol lysates from PBS or rHsCTSL-treated cells, stained for SNCA using the polyclonal proteintech antibody. Quantification of **(M)** soluble SNCA normalized to ACTB or CBB (n = 3). **(N)** Western blot of T-insol lysates after rHsCTSL treatment. Quantification of total **(O)** insoluble SNCA signal positive for SNCA polyclonal antibody normalized to CBB (n = 3). **(P)** Representative Western blot of T-sol lysates from cells treated with PBS, rHsCTSB, or rHsCTSL combined with 0.2 µM Baf for up to 72 h, stained for SNCA. **(Q)** Quantification of insoluble SNCA normalized to CBB (n = 3).. All data represent mean ± SEM. Statistical analyses were performed using one-way ANOVA with Dunnett’s multiple comparison test relative to PBS treatment. ***p<0.001, **p < 0.01, *p < 0.05, n.s. = not significant

To further investigate the role of CTSB and CTSL in SNCA degradation, we utilized a doxycycline-repressible (tet-off) H4 neuroglioma cell line with SNCA overexpression (H4 SNCA) to mimic pathophysiological conditions. H4 cells were treated with 20 µg/mL of rHsCTSB or rHsCTSL for 24, 48, and 72 h. Cell lysates were sequentially extracted using ultracentrifugation and separated into Triton-soluble (T-sol) and Triton-insoluble (T-insol)/ SDS-soluble fractions [54]. This is important to examine the function of the cathepsins on different SNCA conformers distinguishable by their solubility in different solvents, like Triton or SDS [54]. Western blot analyses indicate high endogenous levels of CTSB in H4 cells (Fig. 2A) with a significant increase in the proform (~ 45 kDa), single chain (~ 33 kDa) and heavy chain (~ 28 kDa) after 72 h of rHsCTSB treatment (Fig. 2A - 2D). Interestingly, treatment with the recombinant enzyme does not significantly affect soluble SNCA levels (Fig. 2A and 2E). However, in line with the significant increase in the active forms of CTSB (Fig. 2A, 2C and 2D), a clear decrease of SNCA in T-insoluble/ SDS-soluble fraction was visible after 72 h (Fig. 2F and 2G), in this SNCA overexpressing cell model. Upon Western blot analysis of H4 cells treated with rHsCTSL, we observed a significant increase in all three forms of CTSL: proform (~ 38 kDa), single chain (~ 30 kDa) and heavy chain (~ 23 kDa) (Fig. 2H − 2K). Treatment with rHsCTSL had no significant impact on SNCA in T-soluble fractions (Fig. 2L − 2M), however, a significant reduction in T-insoluble/ SDS-soluble SNCA was achieved 72 h post-treatment. (Figure 2N and 2O). To confirm that these effects are due to the proper maturation and lysosomal activity of the enzymes, H4 SNCA cells were treated with Bafilomycin A1 (Baf), affecting lysosomal function by inhibition of the vacuolar-type H^+^-ATPase. Cells were then treated with either 20 µg/mL rHsCTSB, rHsCTSL or the combination of enzyme along with 0.2 µM Baf for up to 48 h. In the Baf-treated cells, no changes in the levels of insoluble SNCA (Fig. 2P and 2Q) could be observed, demonstrating that insoluble SNCA proteolysis by the recombinant enzymes requires functioning lysosomes.

Overall, our findings confirm uptake and maturation of rHsCTSB and rHsCTSL in H4 cells overexpressing SNCA, and show the reduction of SNCA in T-insoluble fraction after treatment with both enzymes respectively in the presence of functional lysosomes.

### rHsCTSB and rHsCTSL treatment decreases various SNCA conformers in PD-DA-iPSn

To confirm our findings in the H4 cells in a more relevant cell model, we investigated the degradative effect of both cathepsins on pathology-associated SNCA in PD patient-derived human iPSC-derived dopaminergic (DA) neurons expressing the SNCA point mutation p.A53T together with the isogenic corrected iPSC line (A53T corr.) [50]. Dopaminergic differentiation was verified by IF analysis of neurons using tyrosine hydroxylase (TH) in the SNCA A53T mutant as well as the isogenic control line (Figure S3A). As treatment of iPSC-derived DA neurons (DA-iPSn) with 10 µg/mL rHsCTSB for 17–18 days did neither show efficient uptake and maturation of the enzyme (Figure S3B-D) nor significant effects on SNCA level (Figure S3B-G), cells were treated 21–25 days with 10 µg/mL of the respective recombinant enzyme, as this concentration also did not reveal cellular toxicity (Figure S3H).

**Fig. 3:**
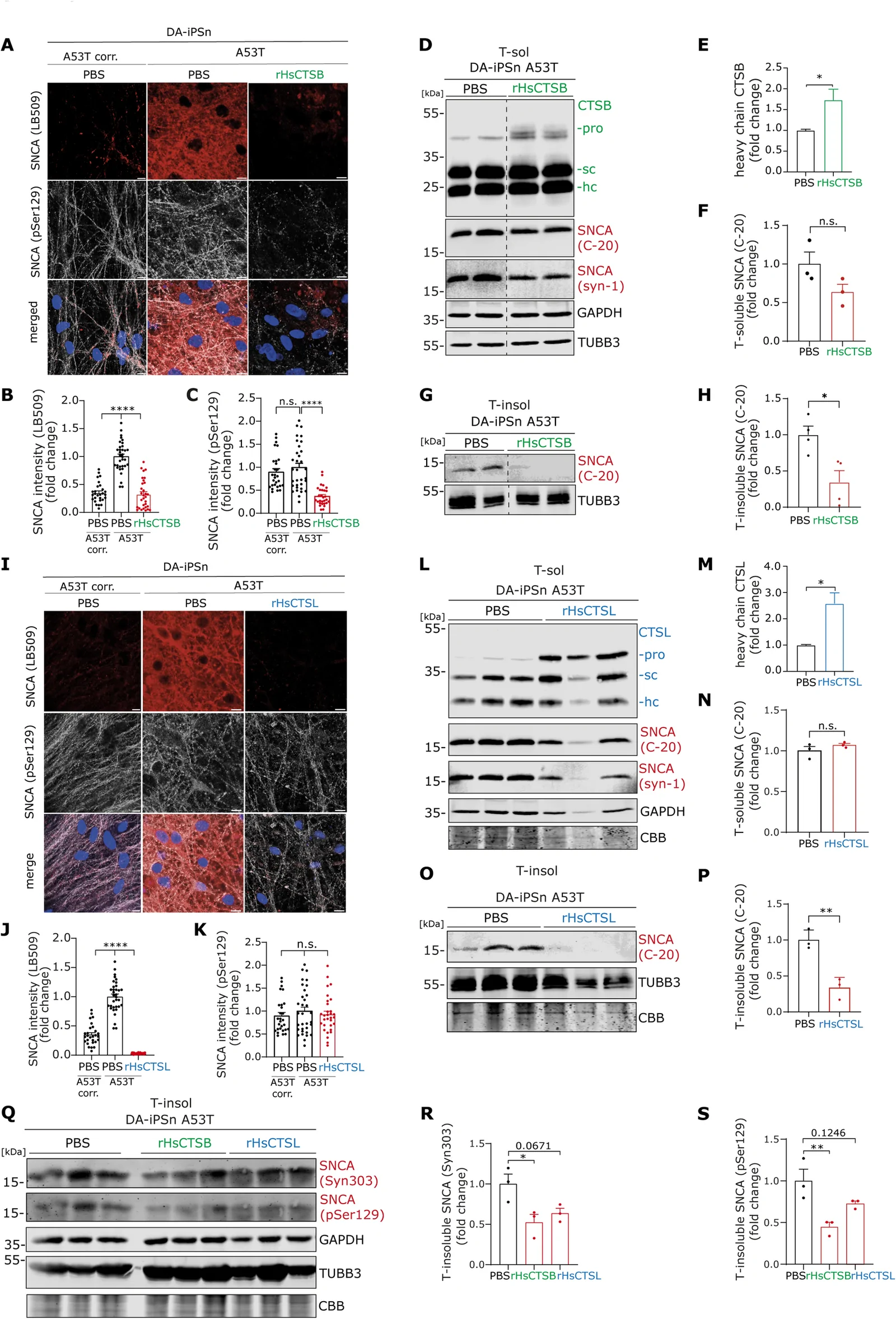
**Treatment with rHsCTSB and rHsCTSL reduces multiple SNCA conformers in PD patient-derived DA-iPSn. (A)** Representative IF images of DA-iPSn A53T mutant and corrected cells stained for pathological SNCA with LB509 (red) and pSer129 (grey) antibodies. Quantification of SNCA intensity for **(B)** LB509 and **(C)** pSer129 was performed and expressed as fold change to A53T mutant (n = 3 biological replicates, 8–12 images/group). **(D)** Western blot of Triton-soluble (T-sol) lysates from DA-iPSn A53T cells treated with PBS or 10 µg/mL rHsCTSB for 21–25 days, stained for SNCA (C-20 and syn-1 antibodies). GAPDH and TUBB3 served as loading controls. Quantification of **(E)** CTSB heavy chain and **(F)** soluble SNCA (C-20) was normalized to GAPDH and expressed as fold change (n = 3). **(G)** Representative Western blot of T-insol./ SDS sol. lysates from rHsCTSB-treated cells, with quantification of **(H)** T-insol./ SDS-sol. SNCA normalized to TUBB3 (n = 4). **(I)** IF images of DA-iPSn A53T mutant and corrected cells treated with rHsCTSL and stained for LB509 (red) and pSer129 (grey). The same corrected DA-iPSn and PBS treated A53T DA-iPSn were used for the analyses in **(A-C)** and **(I-K)**. Quantification of SNCA intensity from** (J)** LB509 and **(K)** pSer129 was performed (n = 3 biological replicates, 8–12 images/group). **(L)** Representative Western blot of T-sol lysates from rHsCTSL-treated cells stained for SNCA (C-20 antibody). GAPDH and CBB were used as loading controls. Quantification of **(M)** CTSL heavy chain and **(N)** soluble SNCA **(C-20)** was normalized to GAPDH (n = 3). **(O)** Representative Western blot of T-insol lysates from rHsCTSL-treated cells, with quantification of **(P)** T-insol SNCA normalized to TUBB3 (n = 3). **(Q)** Representative Western blot of T-insol lysates treated with rHsCTSB or rHsCTSL stained for Syn303 and pSer129. Full-length blots containing lower molecular bands for SNCA shown in Supplementary file-2 along with the analysis on the different forms of SNCA. Quantification of SNCA by **(R)** Syn303 and **(S)** pSer129 normalized to CBB (n = 3). All data represent mean ± SEM. Statistical analyses were performed using one-way ANOVA with Dunnett’s multiple comparison test **(B, C, J, K, R, S)** or a two-tailed unpaired Student’s t-test** (E, F, H, M, N, P) **relative to PBS treatment. ***p<0.001, **p < 0.01, *p < 0.05, n.s. = not significant

This allowed us to assess potential long-term treatment effects by utilizing imaging and Western blot analyses. For IF, A53T DA-iPSn were stained for SNCA with two antibodies detecting different conformers of SNCA: LB509 (red) and SNCA pSer129 (grey) (Fig. 3A). While LB509 was generated against pathology-associated SNCA present in Lewy bodies (LB), hence exhibiting higher affinity towards pathological than physiological SNCA [60, 61], the pSer129 antibody detects phosphorylation of SNCA at serine 129, which is a posttranslational modification of the protein and also found in deposits of LBs [62]. However, this protein modification was also recently linked to physiological SNCA function [63, 64]. Treatment of A53T DA-iPSn with rHsCTSB resulted in a more than two-fold reduction in SNCA levels, as demonstrated by the use of two SNCA antibodies LB509 and pSer129 (Fig. 3A - 3C). After rHsCTSB treatment, SNCA levels detected by LB509 in A53T DA-iPSn decreased to levels of the corrected control line. As pSer129 SNCA signals were prominent in the corrected line, rHsCTSB treatment reduced the level of this SNCA form below control level (Fig. 3A - 3C).

Analysis of DA-iPSn A53T by Western blot also showed high endogenous levels of the single- and heavy chain of CTSB (Fig. 3D and 3E). Treatment of 21–25 days with rHsCTSB significantly increased the levels of the active heavy chain of CTSB, confirming that the recombinant enzyme was efficiently taken up and matured in lysosomes (Fig. 3D and 3E). For detailed analyses of SNCA, a sequential extraction protocol of the cell lysate was applied [54] in combination with various SNCA antibodies: C-terminal: C-20, total: syn-1, pathology-associated: Syn303 (also N-terminal) and pSer129. Although, no changes in T-soluble SNCA protein levels using C-20 and syn-1 antibody were observed (Fig. 3D and 3F), the amount of SNCA in T-insoluble/ SDS-soluble fraction was significantly reduced utilizing C-20 (Fig. 3G and 3H). To validate these effects, we complemented our findings by using an alternative three step-sequential extraction protocol [55], generating three distinct SNCA fractions: TBS/Triton-soluble, RIPA-soluble, and Urea/SDS-fraction. SNCA levels were assessed by Western blot using syn-1 SNCA antibody. In the TBS/Triton-soluble fraction, SNCA levels did not differ significantly between A53T mutant, A53T corrected, or rHsCTSB-treated cells (Figure S3I, S3J). In the RIPA-soluble fraction, we observed a marked increase in SNCA in A53T mutant cells compared to the corrected line, which was significantly reduced upon rHsCTSB treatment (Figure S3K, S3L). In the Urea/SDS-soluble fraction, robust SNCA signal was detected in the A53T mutant line, but was absent in the corrected line and strongly reduced after rHsCTSB treatment (Figure S3M). Notably, these findings using this three-step approach are consistent with our primary extraction method, and further support the robustness of the treatment effects on SNCA in T-insoluble/ SDS-soluble fractions.

Treatment of DA-iPSn A53T with rHsCTSL was carried out similar to rHCTSB using 10 µg/mL for 21–25 days. IF analyses indicate a significant reduction in SNCA stained with LB509 antibody, but interestingly no effect on the phosphorylated form of SNCA (pSer129) was observed (Fig. 3I and 3K). Uptake and maturation of rHsCTSL was confirmed by Western blot analysis which revealed a significant increase in levels of the active heavy chain form of CTSL (Fig. 3L and 3M). Similar to the effect of rHsCTSB treatment (Fig. 3D and 3F), no changes in soluble SNCA were observed after treatment with rHsCTSL (Fig. 3L and 3N). Remarkably, more than half of insoluble SNCA detected by C-20 antibody was decreased after incubation with the cathepsin (Fig. 3O and 3P). To further validate our findings on the reactivity of the two recombinant enzymes towards different SNCA species, Western blot analyses were carried out using additional antibodies against pathology-associated, posttranslational-modified SNCA forms: Syn303 (targeting oxidized and nitrated SNCA [65]) and the above-used pSer129 (Fig. 3Q). Both, rHsCTSB as well as rHsCTSL treatment, resulted in a reduction in oxidized and nitrated SNCA (*p* < 0.05 for rHsCTSB and *p* = 0.0671 for rHsCTSL) (Fig. 3R). In line with our IF analysis, we observed a significant reduction in phosphorylated forms of SNCA of around 50%, after treatment with rHsCTSB, while rHsCTSL treatment showed a decreasing but non-significant trend (*p* = 0.1246) (Fig. 3S). To investigate effects of the treatment on pathology-associated forms of SNCA, Western blot analysis using higher protein input (40 µg) revealed several lower molecular weight bands reactive to specific SNCA antibodies (Syn303, pSer129). Full-length, uncropped blots for all experiments are provided in Supplementary File 2. Notably, analysis of these lower molecular weight bands revealed no increase in truncated SNCA species following treatment with recombinant cathepsins; instead, a consistent reduction in their levels was observed (Supplementary File 2).

As control and to examine whether the intraneuronal response to cathepsin administration is influenced by SNCA pathology present in neurons, we also treated the isogenic corrected (corr.) DA-iPSn line with both recombinant enzyme. The corresponding analyses conducted in the A53T corr. line revealed rHsCTSB uptake, depicted by the increased protein levels of proCTSB (Figure S3N, S3O). Interestingly, significant differences could only be observed in the proform of the enzyme, but not in the single or heavy chain of CTSB nor in soluble SNCA levels (Figure S3N – S3R). This suggests a reduced requirement for active cathepsin within the lysosome in the control line, presenting without SNCA pathology indicated by the absence of SNCA protein signal in the T-insoluble/ SDS-soluble fractions (Figure S3S). The same set of Western blot analyses was carried out for rHsCTSL treatment in the corr. A53T control line (Figure S3T). All forms of CTSL (pro-, single chain and heavy chain) were significantly increased (Figure S3T – S3W), while no changes were observed on soluble SNCA between treated and non-treated cells (Figure S3T, S3X). No SNCA signal was detected in the control line in T-insoluble/ SDS-soluble fraction indicating the absence of SNCA pathology before and after rHsCTSL treatment (Figure S3Y).

Taken together, our data from PD-DA-iPSn harboring SNCA A53T mutation, underscore the effects of rHsCTSB and rHsCTSL treatment on clearance of various forms of SNCA, as found in T-insoluble/ SDS-soluble fractions as well as by using pathology-preferable SNCA antibodies (Syn303, pSer129, LB509).

### No synergistic effects of rHsCTSB/rHsCTSL combination treatment on SNCA degradation, but improvement of lysosomal function

**Fig. 4:**
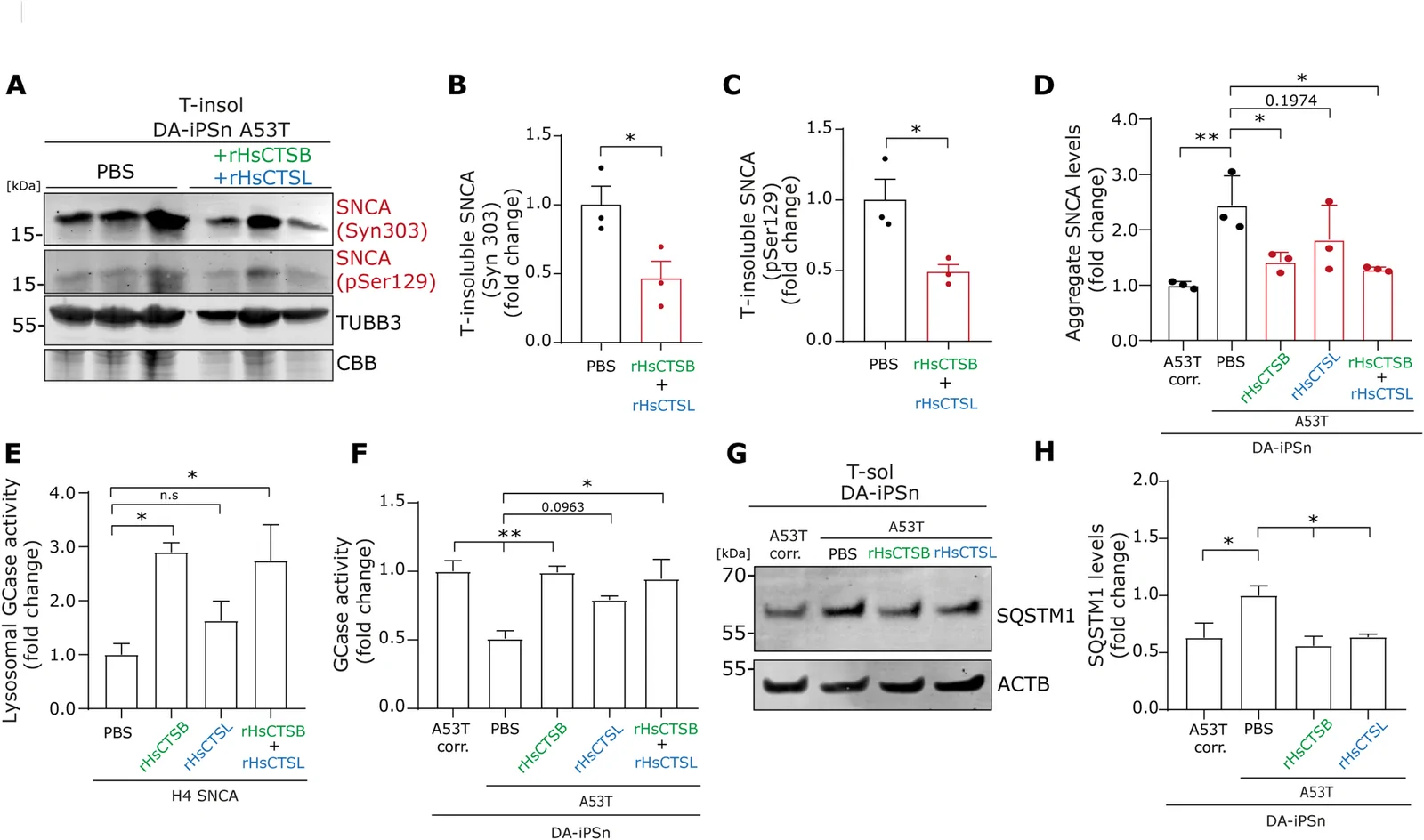
**Effects of individual and combined treatment of rHsCTSB and rHsCTSL on SNCA clearance and lysosomal-autophagic functions in PD DA-iPSn.** **(A)** Representative Western blot of T-insol lysates from DA-iPSn A53T treated with PBS or a combination of 10 µg/mL of rHsCTSB and rHsCTSL. Blots were stained for pathology associated forms of SNCA using Syn303 and pSer129 antibodies. Quantification of Triton-insoluble/SDS-soluble (T-insol) SNCA stained with **(B)** Syn303 and **(C)** pSer129 was normalized to TUBB3 and expressed as fold change (n = 3). **(D) **Analysis of SNCA aggregate ELISA showing levels of SNCA aggregates within the DA-iPSn A53T with PBS or 10 µg/mL rHsCTSB, rHsCTSL or the combination of rHsCTSB and rHCTSL treatment and expressed as fold change to A53T corrected line (n = 3). **(E)** Lysosomal GCase activity was measured in H4 cells treated with 20 µg/mL rHsCTSB, rHsCTSL, or the combination of both enzymes (n = 4) and expressed as fold change to PBS treatment. **(F) **GCase activity was measured in the cell lysate of DA-iPSn, treated with rHsCTSB, rHsCTSL or the combination of rHsCTSB and rHCTSL, and expressed as a fold change to A53T corrected line (n = 3). **(G)** Representative Western blot from DA-iPSn treated with PBS or 10 µg/mL of rHsCTSB and rHsCTSL, stained for SQSTM1/p62. **(H)** Quantification of SQSTM1 levels normalized to ACTB and expressed as fold change (n = 3) to PBS treated A53T mutant line. All data represent mean ± SEM. Statistical analyses were performed by using a two-tailed unpaired Student’s t-test **(B, C)** or one-way ANOVA together with Dunnett’s multiple comparison test **(D, E, F, H)**. Statistical differences are shown toward PBS treated A53T mutant cells. ****p < 0.0001, ***p < 0.001, **p < 0.01, *p < 0.05; n.s., not significant

Since both, CTSB and CTSL showed to be able to degrade pathology-associated forms of SNCA, we next tested the effect and efficiency of both CTSB and CTSL in combination regarding SNCA degradation. For this, DA-iPSn A53T were treated as described previously for 21–25 days with 10 µg/mL rHsCTSB and 10 µg/mL rHsCTSL. To confirm uptake and maturation of both recombinant enzymes, Western blots were stained for CTSB (Figure S4A- S4D) and CTSL (Figure S4E – S4H). A significant increase was demonstrated in the proform and mature heavy chain form of both cathepsins when treated in combination. To analyze the effect in DA-iPSn A53T neurons after the combinational treatment with both enzymes (rHsCTB and rHsCTSL), Western blot analyses revealed a significant decrease in SNCA levels in the T-insoluble/ SDS-soluble protein fraction utilizing Syn303 and pSer129 SNCA antibodies (Fig. 4A- 4C). To better compare between different application strategies (individual and combined treatment) and to evaluate the rescue efficiency within one analytical approach, we analyzed whole lysates of iPSC-derived DA neurons by an ELISA, specific for aggregated SNCA. Treatment with rHsCTSB as well as the combined treatment resulted in significant reduction of aggregated SNCA compared to untreated A53T mutant iPSn-DA neurons (Fig. 4D). Treatment with rHsCTSL showed a decreasing, but non-significant trend in aggregated SNCA form as measured by the ELISA (*p* = 0.1974 for rHsCTSL) (Fig. 4D). In parallel, we investigated the effects of the treatment using an ELISA measuring total SNCA amount (no specificity for pathological conformers). Interestingly, the only significant difference was found between the A53T mutant and corrected control (Figure S4I). Treatment with individual proteases as well as the combination of both had no significant effect on the total SNCA levels (Figure S4I) underlining the effects of both cathepsins on pathology-associated SNCA conformers.

Next, effects of indvidual as well as combined treatments on lysosomal and autophagy function was evaluated by analyzing GCase activity as well as by analyzing protein level of the autophagy marker SQSTM1/p62. Interestingly, in the H4 SNCA overexpressing model, the lysosomal activity of the PD-associated enzyme GCase was increased after treatment with rHsCTSB and in combinantion with both enzymes (Fig. 4E). Treatment with rHsCTSL, led to a moderate, but non-significant increase in lysosomal GCase activity (Fig. 4E). Consistently, in the DA-iPSn, an improvement in GCase activity in the rHsCTSB and combined treated A53T neurons similar to control corrected line, as compared to untreated cells was observed (Fig. 4F). Treatment with rHsCTSL led to a statistically not significant increase in GCase activity (*p* = 0.0963; Fig. 4F). Treatment with both rHsCTSB and rHsCTSL recovered SQSTM1/p62 levels in treated A53T mutant cells to healthy control (A53T corr.) level (Fig. 4G and H).

Taken together, our data indicate that the combined treatment with rHsCTSB and rHsCTSL contributes to a reduction in pathology-associated SNCA forms. Furthermore, treatment with the individual enzymes as well as a combination of both improved endolysosomal function. However, no clear evidence of a synergistic effect of both enzymes administered together could be observed on SNCA degradation or on lysosomal function.

### Administration of rHsCTSB and rHsCTSL to organotypic brain slices and primary neurons from Thy1-SNCA mice reduces SNCA pathology and SNCA-dependent neurotoxicity

**Fig. 5:**
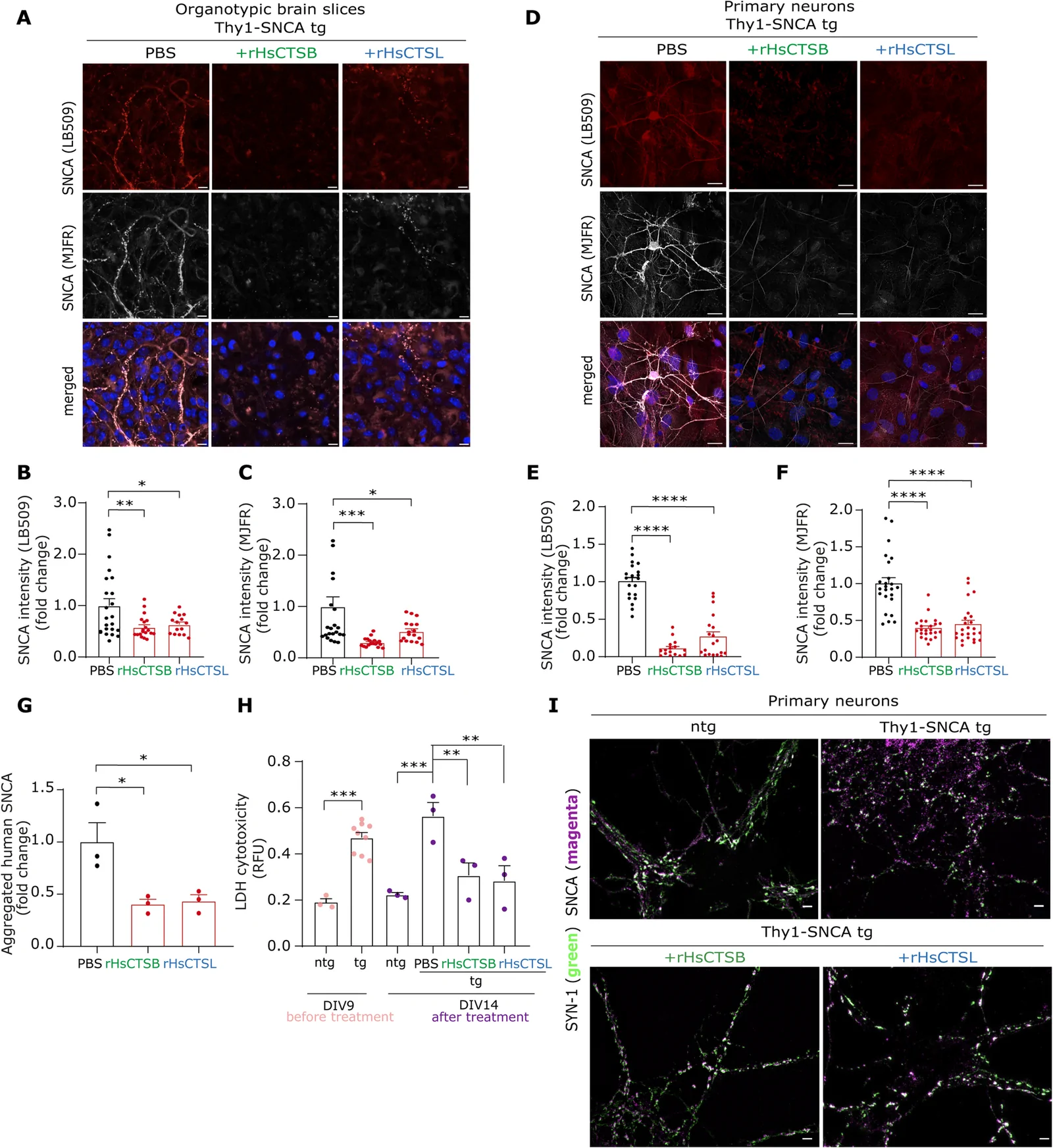
**rHsCTSB and rHsCTSL treatment reduces pathology-associated SNCA in organotypic brain slices and primary neurons from Thy1-SNCA overexpressing mice.** **(A)** Representative IF images of brain slices from transgenic (tg) postnatal day 10 Thy1 mice. Slices were treated with either 20 µg/mL rHsCTSB or rHsCTSL or the same volume of PBS for two weeks and stained with pathology-associated SNCA antibodies LB509 (red) and MJFR-14-6-4-2 (grey). Quantification of SNCA signal intensity of whole picture area after treatment (n = 3 treated brain slices, 6–8 pictures per slice) using **(B)** LB509 or **(C)** MJFR-14-6-4-2, Scale bar: 10 μm. **(D)** Representative IF images of primary neurons from Thy1 mice treated with rHsCTSB or rHsCTSL stained with pathology-associated SNCA antibodies LB509 (red) and MJFR-14-6-4-2 (grey). DAPI staining shown in blue. Scale bar: 20 μm. Quantification of SNCA signal intensity of whole picture area after treatment (n = 3, primary neurons from 3 different Thy1-SNCA mice per group, 6–8 pictures per coverslip,) using **(E)** LB509 (red) or **(F)** MJFR-14-6-4-2 (grey). **(G)** Analysis of SNCA aggregate ELISA in the primary neurons from Thy1-SNCA tg mice treated with rHsCTSB and rHsCTSL and expressed as fold change to the PBS treated control. (n = 3). **(H)** LDH assay for non-transgenic (ntg) and Thy1-SNCA transgenic (tg) primary neurons, at DIV 9 before the start of treatment and DIV 14, after 4 days of treatment with rHsCTSB or rHsCTSL. (n = 3, primary neurons from 3 different mice, per condition). **(I)** Representative structured illumination microscopy (SIM) images providing detailed localization of SNCA to Synapsin-1 (SYN-1) in primary neurons treated with rHsCTSB and rHsCTSL. Neuronal processes were immunostained with SNCA antibody syn-1 (magenta) and Synapsin-1, SYN-1 (green). Scale bar: 2 μm. All data represent mean ± SEM. Statistical analyses were performed by using one-way ANOVA followed by Dunnett’s **(B, C, E, F, G)** or Tukey’s post hoc **(H)** for multiple comparisons. Statistical differences are shown toward PBS treated slice/neuron from transgenic mice. ****p < 0.0001, **p < 0.01, *p < 0.05; n.s., not significant

To further validate the proteolytic effect of rHsCTSB and rHsCTSL within a PD-relevant model, we utilized a well-characterized PD mouse model overexpressing human SNCA driven by the murine Thy1 promoter. The heterozygous transgenic mice demonstrate loss of striatal dopamine, SNCA pathology [43, 44, 68, 69], and progressive motor deficits, the major characteristics of PD [43, 44, 70]. We prepared cortical brain slices from 10-day-old transgenic mice and treated them with 20 µg/mL rHsCTSB or rHsCTSL for two weeks (experimental setup: Figure S5A) every other day. Brain slices were stained with two different SNCA antibodies: LB509 (red) and MJFR-14-6-4-2 (grey), a conformer-specific antibody against fibrillar SNCA [71]. Confocal imaging of non-treated brain slices (Thy1 tg PBS) revealed high SNCA signal intensity, confirming the presence of SNCA pathology in this novel ex vivo model (Fig. 5A). Treatment with rHsCTSB and rHsCTSL significantly reduced SNCA pathology, showing strong reduction in LB509 as well as MJFR-positive SNCA. (Fig. 5A-5C). Uptake of rHsCTSB and rHsCTSL was confirmed by IF stainings (Figure S5B). Further, co-staining of the neuronal marker neurofilament and SNCA antibody MJFR, confirmed the co-localization of pathological SNCA with neurons (Figure S5C). To complement our findings from the ex vivo model, we utilized primary neurons from the same model to further validate treatment effects in a neuron-enriched environment. Cortical primary neurons were generated from postnatal day 0 or day1 Thy1-SNCA transgenic (tg) as well as non-transgenic (ntg) mice and the neurons were treated with rHsCTSB or rHsCTSL for 5 days. To confirm uptake and maturation of the recombinant pro-enzymes, lysate activity assays confirmed increased CTSB as well as CTSL activity after treatment (Figure S5D, S5E). SNCA pathology in the primary neurons was validated using the IF staining for LB509 and MJFR antibodies, revealing high levels of pathology-associated SNCA in neurons derived from Thy1 SNCA tg mice, compared to ntg control (Figure S5F). Treatment with rHsCTSB as well as rHsCTSL led to a significant decrease in SNCA levels recognized by both LB509 and MJFR antibodies in IF analyses (Fig. 5D - 5F). To quantitatively assess the impact of the treatment on SNCA, ELISA experiments were performed in primary neurons of Thy1-SNCA tg animals. rHsCTSB as well as rHsCTSL treatment led to significant reduction in aggregated human SNCA (Fig. 5G). Analyzing total human SNCA amounts by ELISA, a significant reduction was observed following rHsCTSB treatment, while rHsCTSL showed a strong trend towards reduction (*p* = 0.0569; Figure S5G). Importantly, the ratio of aggregated to total SNCA, a proxy for aggregation burden, was significantly decreased in both treatment groups, indicating a shift towards a less aggregated SNCA profile in the model (Figure S5H). To investigate effects of the treatment on cell toxicity, an LDH assay was carried out in this model. At DIV9, before the start of the treatment, we observed a significant baseline difference in cytotoxicity in the primary neurons derived from Thy1-SNCA tg and ntg mice (Fig. 5H). Interestingly, at DIV14, after 4 days of treatment with rHsCTSB as well as rHsCTSL, a significant reduction in cell toxicity compared to untreated neurons from the same transgenic mouse was observed (Fig. 5H). Consistent with the LDH assay data, immunostaining for the neuronal marker TUBB3 revealed an increased number of TUBB3-positive extensions after treatment (Figure S5I), further supporting an early neuroprotective effect of both lysosomal cathepsins. Next, synaptic homeostasis was analyzed in our primary neuronal model by co-staining for Synapsin-1 (SYN-1) and the SNCA antibody (syn-1), recognizing both, murine and human SNCA. Using Structured Illumination Microscopy (SIM), differences in intensity, overlap with SNCA and distribution of synapsin-1 in neurons from transgenic Thy1-SNCA mice compared to the non-transgenic control were observed (Fig. 5I). These effects could be reversed after CTSB and CTSL treatment (Fig. 5I).

Collectively, these findings demonstrate the capacity of rHsCTSB and rHsCTSL to reduce SNCA burden in an ex vivo as well as in vitro primary neuronal system derived from a well-characterized PD mouse model. Additionally, SNCA-mediated neurotoxicity and changes in TUBB3 as well as Synapsin-1 distribution were ameliorated, further underlining their therapeutic potential.

### *In vivo* treatment of rHsCTSB and rHsCTSL decreases SNCA in Ctsd-deficient mice

**Fig. 6:**
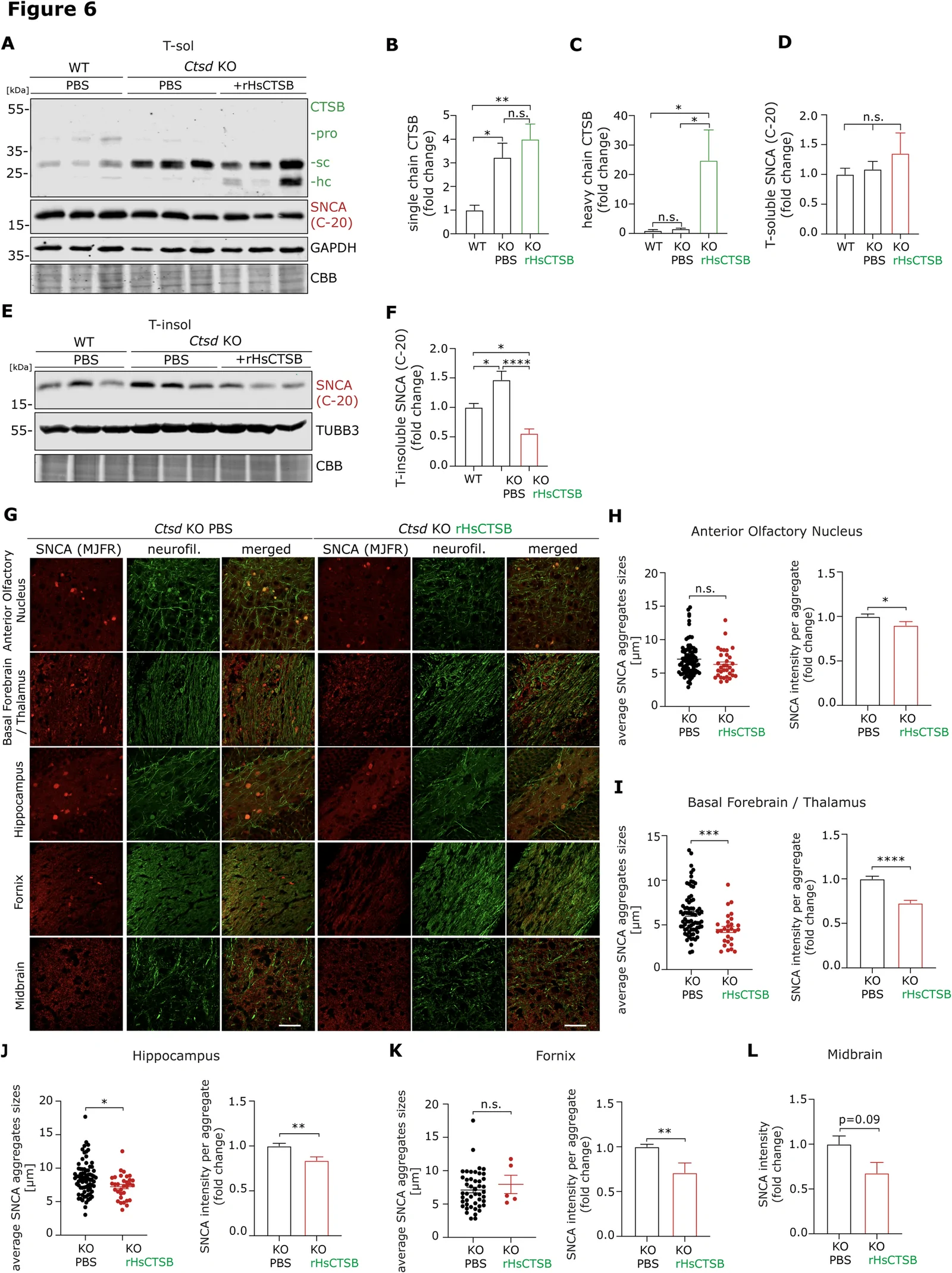
**Proof-of-concept in vivo study: intracranial injection of rHsCTSB decreases SNCA in the brain of *CTSD *deficient (KO) mice.** **(A)** Representative Western blot of Triton-soluble (T-sol) lysates from WT and Ctsd KO mice brains injected with PBS or 100 µg rHsCTSB at P1 (left hemisphere) and P19 (right hemisphere). For CTSD and SNCA (syn-1) stainings, see Figure S6A. Quantification of **(B)** the single chain, **(C)** heavy chain of CTSB, and **(D)** soluble SNCA (C-20; syn-1 in Figure S6B) was normalized to TUBB3 and expressed as fold change (n = 3 mice/group). **(E)** Western blot of Triton-insoluble/SDS-soluble (T-insol) lysates from KO brains with or without rHsCTSB treatment. For SNCA (syn-1) staining, refer to Figure S6C. Quantification of **(F)** T-insol SNCA (C-20; syn-1 in Figure S6D) was normalized to TUBB3 and expressed as fold change (n = 3 mice/group). **(G)** Representative images of brain regions (anterior olfactory nucleus, basal forebrain/thalamus, hippocampus, fornix, and midbrain) from KO and KO + rHsCTSB mice co-stained with conformation-specific SNCA antibody MJFR-14-6-4-2 (red) and neuronal marker neurofilament (green). Brain region analysis is outlined in Figure S6E. Scale bar: 50 μm. SNCA aggregates analysis: Size and intensity of SNCA aggregates were measured in **(H)** anterior olfactory nucleus, **(I)** basal forebrain/thalamus, **(J)** hippocampus, **(K) **fornix, and **(L)** SNCA intensity in midbrain (n = 3 mice/group). Dots represent SNCA aggregates per image in the analyzed regions. All data represent mean ± SEM. Statistical analyses were conducted using one-way ANOVA with Tukey’s test **(B–D, F)** or two-tailed unpaired Student’s t-test **(H–L)**. Statistical differences are shown relative to PBS treatment. ****p < 0.0001, ***p < 0.001, **p < 0.01, *p < 0.05; n.s., not significant

As both, rHsCTSB and rHsCTSL appear to enhance the clearance of pathology-associated SNCA species as well as neuronal homeostasis and survival in vitro, we next investigated the therapeutic potential in vivo. For this proof-of-concept study, we used the extensively characterized *Ctsd* KO mouse model- which illustrates the neuropathology of human neuronal ceroid lipofuscinosis type 10- given the presence of prominent SNCA aggregation in the brain [45, 66]. This mouse model shows severe dysfunctions in the autophagy-lysosomal system indicated by accumulations of autophagosomes or a disturbed degradative capacity [47]. Previous studies using this model have established a robust treatment paradigm - including optimized dosing and safety evaluations of the recombinant cathepsins - making it a suitable and validated model for investigating preliminary therapeutic effects in vivo [52]. *Ctsd* KO mice were intracranially injected (i.c.) in both hemispheres with 100 µg (in 10 µL PBS) of rHsCTSB at postnatal day 1 (P1, right hemisphere) and at P19 (left hemisphere). All studied mice were sacrificed at P23 given their drastic decline in health in the homozygous mice used in our experiment [66]. Western blot analyses of whole brain lysates revealed the presence of pro- (~ 52 kDa) and mature forms (heavy chain, ~ 34 kDa) of CTSD in wildtype (WT) mice and the absence of CTSD protein in *Ctsd* KO mice (Figure S6A). Elevated levels of the single chain form of CTSB (~ 33 kDa) were observed in *Ctsd* KO mice compared to WT (Fig. 6A and 6B). We speculate this to reflect a compensatory mechanism due to the lack of CTSD, one of the main hydrolases for lysosomal function. While almost no proCTSB was detected in *Ctsd* KO mice injected with rHsCTSB, we observed an increase in the heavy chain form (~ 28 kDa) (Fig. 6A and 6C), suggesting a rapid turnover of the recombinant enzyme. Soluble SNCA levels remained unchanged between WT, KO and KO + rHsCTSB mice using the C-20 (Fig. 6A and 6D) or syn-1 SNCA antibody (Figure S6A- S6B). However, T-insoluble/ SDS-soluble brain lysates showed high SNCA levels in *Ctsd* KO mice (Fig. 6E), as previously demonstrated [16]. Mice that were i.c. injected with rHsCTSB exhibited significantly lower insoluble SNCA, revealed by the C-20 antibody (Fig. 6E and 6F). While statistical significance was not achieved using the syn-1 antibody in T-insoluble/ SDS-soluble fractions under CTSD deficiency (*Ctsd* KO) as well as after rHsCTSB treatment (Figure S6C- S6D).

To further validate the CTSB proteolysis of insoluble SNCA, we investigated SNCA levels in brain slices by IF, utilizing the conformation-specific MJFR-14-6-4-2 antibody against pathological (fibrillar) SNCA [67]. The analyzed mouse brain regions, including the anterior olfactory nucleus, the region between the basal forebrain and thalamus, hippocampus, fornix, and midbrain, are illustrated in a schematic representation of a sagittal cut (Figure S6E). Brain slices were co-stained with neurofilament as a neuronal marker in *Ctsd* KO mice (Fig. 6G) and WT mice (Figure S6F). SNCA aggregates, defined by dense bodies with high SNCA intensity, were observed in the anterior olfactory nucleus (mean average size: 7.07 µm ± 0.4), in the area between the basal forebrain and thalamus (mean average size: 6.25 μm ± 0.5), hippocampus (mean average size: 8.53 μm ± 0.4), and fornix (mean average size: 7.11 μm ± 1.3) of *Ctsd* KO mice (Fig. 6G). A decrease of SNCA aggregates was observed after rHsCTSB treatment in the anterior olfactory nucleus (Fig. 6H), albeit no significant changes in the mean average size of SNCA aggregates (6.29 μm ± 0.7). rHsCTSB-treated mice also present smaller SNCA aggregates in the area between the basal forebrain and thalamus (mean average size: 4.49 μm ± 1.7), and hippocampus (mean average size: 7.24 μm ± 1.2), as well as less SNCA immunopositivity per aggregate (Fig. 6I and  6J). No statistical difference was determined between the size of SNCA aggregates in the fornix (mean average size: 7.95 μm ± 0.8), a decrease in SNCA signal intensity was revealed (Fig. 6K). Furthermore, IF staining indicates pathological accumulation of SNCA in the midbrain, which was reduced following treatment with rHsCTSB (*p* = 0.09) (Fig. 6L). Quantification of SNCA aggregates per mm² in *Ctsd* KO mice showed no significant difference in the anterior olfactory nucleus following rHCTSB treatment (Figure S6G). However, there was a significant decrease in the number of aggregated SNCA within the region between the basal forebrain and thalamus, hippocampus, and fornix after rHsCTSB treatment (Figure S6H- S6J).

To validate the proteolytic effect of CTSL on pathological SNCA *Ctsd* KO mice, animals were i.c. injected with PBS (KO PBS) or 100 µg (in 10 µL PBS) rHsCTSL (KO rHsCTSL) at P1 and P19 and were sacrificed at P23 similar to the treatment with rHsCTSB above. In this proof-of-concept study, brain slices of KO PBS and KO rHsCTSL mice were costained with SNCA and neurofilament antibodies (Figure S7A) and analyzed as described for *Ctsd* KO mice injected with rHsCTSB (Fig. 6G and 6). The same KO PBS mice analyzed in Figs. 6G and 6L were used to evaluate KO rHsCTSL animals. Treatment with rHsCTSL resulted in a significant decrease of SNCA intensity within aggregates found in the anterior olfactory nucleus (Figure S7B), the region in between the basal forebrain and thalamus (Figure S7C) the hippocampus (Figure S7D) and the fornix (Figure S7E). Additionally, rHsCTSL administration led to a reduction in the size of SNCA aggregates in the hippocampus (mean average size: 7.2 μm ± 1.33) and fornix (mean average size: 4.7 μm ± 2.4), but not in the anterior olfactory nucleus (mean average size: 6.5 μm ± 0.47) or basal forebrain/thalamus (mean average size: 5.6 μm ± 0.57). In addition, an overall decrease in SNCA intensity was observed in the midbrain of KO rHsCTSL mice (Figure S7F). A reduction in the number of SNCA aggregates per mm² was shown in the anterior olfactory nucleus (Figure S7G), basal forebrain/thalamus (Figure S7H) and hippocampus (Figure S7I) of *Ctsd* KO mice dosed with rHsCTSL but no changes were observed in the fornix (Figure S7J).

Altogether, our proof-of-concept in vivo data indicate that intracranially applicated rHsCTSB and rHsCTSL promote the clearance of pathology-associated SNCA aggregates within mouse brain and thus should be evaluated in further in vivo studies.

## Discussion

Increasing evidence has highlighted the importance of the autophagic and lysosomal pathway function in neurodegenerative disease [28, 72]. Given their post-mitotic state and limited regenerative potential, neurons are particularly vulnerable to deficits in the autophagy-lysosomal pathway. The normal decline in lysosomal function that accompanies aging further contributes to neuropathology [73]. Thus, enhancing autophagy and lysosomal function represents a promising strategy for tackling neurodegenerative pathologies [74].

We here demonstrate that boosting lysosomal proteases CTSB and CTSL reduce SNCA burden and improve lysosomal/autophagy function in different human and murine models. Cysteine proteases CTSB and CTSL are two widely expressed lysosomal enzymes that play central roles in autophagy, lysosomal function, and a range of other physiological processes [52, 75, 76]. Their functional synergy is highlighted in studies of double-knockout mice, where simultaneous loss of both enzymes leads to pronounced cerebral and cerebellar atrophy, widespread neuronal loss, abnormal accumulation of lysosomes, and ultimately death within three to four weeks after birth [77]. In contrast, mice lacking only CTSB or only CTSL do not display significant neuronal loss, underscoring their compensatory interplay for neuronal survival [78, 79]. Importantly, both enzymes are implicated in the processing of aggregate-prone proteins such as amyloid-beta precursor protein [80–82], linking them to the pathogenesis of neurodegenerative diseases [17]. Furthermore, genetic variants, reduced protein levels, and diminished catalytic activity of CTSB and CTSL have been associated with neuropathology, including Parkinson’s disease [31, 36, 37], highlighting the essential role of both proteases in maintaining neuronal health. Recent studies show that pharmacological and genetic inhibition of CTSB and CTSL result in accumulation of SNCA [34] and reduced glucocerebrosidase activity in cell lines, dopaminergic neurons as well as midbrain organoids [40], further underlining the functional importance of these cysteine proteases. Vice-versa also SNCA pathology has been shown to impact maturation and function of lysosomal cathepsins, indicating a close functional interaction and dependence [34].

To further investigate the catalytic roles of CTSB and CTSL in SNCA degradation, we examined whether enhancing CTSB or CTSL could promote SNCA clearance, applying recombinant enzymes consisting of the human proforms (rHsCTSB/rHsCTSL). This approach resembles the principles of enzyme replacement therapy (ERT), which is the gold standard treatment for many lysosomal storage disorders, like Gaucher’s disease [83]. As shown before [16, 47], the non-active, recombinant procathepsin undergoes maturation and activation in an acidic environment (pH 4.5–5.0) [84, 85], ensuring catalytic activity only within acidic lysosome. Hence, for our treatment strategy we chose the recombinant proforms of the CTSB and CTSL, preventing undesired enzymatic activity outside lysosomes. Importantly, the applied concentrations for rHsCTSB and rHsCTSL did not cause cytotoxicity in any of the models used here throughout the treatment duration.

Multiple studies on Alzheimer’s disease pathology demonstrated anti-amyloidogenic properties of CTSB or CTSL [81, 86, 87] *via* direct [81, 88] or indirect enhancement of its enzymatic activity [87, 89, 90]. McGlinchey and colleagues suggested that CTSB mediates SNCA proteolysis by targeting its central and amyloid regions, whereas CTSL seems to cleave the C-terminal acidic region [15]. Previous in vitro studies indicate that CTSB incompletely cleaves SNCA fibrils, producing C-terminal truncations that accelerate amyloidogenic fibril formation. In contrast, CTSL showed more efficient degradation of SNCA fibrils [15, 39], a finding supported by our own in vitro assays. We observed that both monomeric and fibrillar SNCA are substrates for rHsCTSB and rHsCTSL. While rHsCTSB partially digested fibrillar SNCA, it rapidly degraded monomeric SNCA, whereas rHsCTSL needed longer time for complete monomer degradation in our in vitro analyses.

However, when recombinant enzymes are applied to cells, the proteases operate within a complex lysosomal environment, functioning in concert with other lysosomal enzymes. In our in vitro cell models - including healthy control DA-iPSn - treatment with either cathepsin did not indicate any enhanced amyloid fibril formation/presence of pathological SNCA conformers as a result of incomplete SNCA degradation. In our study, we employed multiple SNCA detection methods - including diverse antibodies and biochemical assays - to characterize various SNCA species (including nitrated/oxidized as well as phosphorylated SNCA forms). Biochemical analyses utilized an established sequential extraction protocol [54, 91, 92] paired with ELISA quantification specific for aggregated or total SNCA level [93]. Importantly, IF analyses enable to also use structure-specific antibodies, like the MJFR-14-6-4-2, which has been shown to be reactive towards pathology-associated SNCA fibrillar structures [71].

The therapeutic potential of both cathepsins was tested in different human and murine models exhibiting SNCA pathology: In human cell models (H4 cells and DA-iPSn), the reduction of pathology-associated SNCA was evaluated via sequential extraction protocol and IF utilizing various SNCA antibodies, as well as an ELISA specific for aggregated SNCA in PD-DA-iPSn. This data in human cells is further supported by the reduction of pathology-associated SNCA species utilizing IF in ex vivo organotypic brain slices as well as SNCA fibril-specific ELISA in primary neurons of a PD mouse model (Thy1-SNCA). Further in vivo application of the enzymes resulted in reduction of pathology-associated SNCA conformer in *Ctsd* KO mouse brain samples.

The important role of CTSB for SNCA degradation as well as lysosomal function was recently supported by another study [40]. Besides the negative impact on pathological SNCA aggregation under CTSB deficiency, they describe positive effects on SNCA clearance as well as on GCase function after CTSB gene activation [40]. Using our recombinant enzyme treatment strategy, our findings align with this pattern: individual as well as combinatorial treatments with recombinant CTSB/CTSL improved lysosomal and autophagy function. This was indicated by enhanced GCase function and reduced SQSTM1/p62 levels in human cells. Critically, decreased p62 - a multifunctional autophagy adapter - signifies enhanced autophagic flux, promoting clearance of toxic protein aggregates implicated in synucleinopathies [94]. Of note, only rHsCTSB treatment resulted in a significant enhancement of GCase activity, whereas rHsCTSL treatment yielded a non-significant trend toward increased GCase activation.

In a recent publication Kim et al. show the importance of CTSL to regulate GCase stability, suggesting a direct degradative role of CTSL towards GCase utilizing an in vitro assay using recombinant proteins [95]. Since treatment with the recombinant enzyme did not impair GCase function, even at the lysosomal level in H4 cells, across both human cell models, we did not see indications for a direct CTSL-dependent GCase degradation in our cellular systems.

Comparing the therapeutic potential of rHsCTSL and rHsCTSB on SNCA, we found less efficient degradation of the pSer129 SNCA form in A53T DA-iPSn for CTSL. As the phosphorylated SNCA was recently described to not only have pathological, but also physiological functions [64], this is an interesting distinction between the role of CTSB and CTSL in SNCA degradation. In A53T DA-iPSn, T-insoluble SNCA species as well as aggregated SNCA indicated by ELISA seemed to be slightly more decreased utilizing rHsCTSB in comparison to rHsCTSL. As rHsCTSL appeared more efficient in clearing fibrillar SNCA forms than rHsCTSB in vitro, this was an unexpected finding. A mechanistic explanation could be that enhanced clearance of monomeric SNCA species prohibits the formation of fibrillar forms as they feed from these lower molecular forms. We described a similar mechanism for the decrease of pathological conformers after treatment with rHsCTSD, which also favored soluble monomer over insoluble fibrils [16]. Further, in vitro degradation assays do not replicate the complex conditions within cells, moreover CTSL has been shown to be less stable outside the lysosomal pH range [96] compared to CTSB [97, 98]. Thus, disrupted pH homeostasis of lysosomes, which might be present under SNCA pathology [99], may contribute to differences between CTSB and CTSL degradation activities in cells harboring synuclein pathology.

To overcome the differential degradation of SNCA by CTSB and CTSL, we co-administered both proteases, with no synergistic degradation effect being observed in the here tested models and conditions. This lack of synergy may be attributable to steric hindrance, where competition for adjacent cleavage sites on the SNCA protein limits concurrent enzymatic activity [15]. To further validate the therapeutic potential of rHsCTSB and rHsCTSL in a pathological setting, we exploited ex vivo organotypic brain slices as well as primary neurons from a PD mouse model. Thy1-SNCA transgenic mice overexpress the human wild-type SNCA under the murine Thy1 promoter (from P10) resulting in widespread SNCA accumulation in different brain areas including the midbrain, cerebellum, cortex, hippocampus, and medulla oblongata [43, 44], recapitulating key features of PD [43].

Organotypic brain slice cultures offer some advantages over in vivo models: They preserve the complexity of the brain structure and synaptic organization of different neural cells without the need for animal treatment [100]. Additionally, they enable a rapid induction of disease-related processes- such as protein aggregation- in a shorter time compared to in vivo models [100–103], underscoring their benefits as an experimental model for neurodegenerative diseases [102]. We further validate our findings in isolated primary neuronal cultures derived from the same mouse model. In both models, SNCA pathology was confirmed by IF analyses utilizing two antibodies targeting different conformers of SNCA: LB509 and the conformation-specific MJFR-14-6-4-2. Treatment with rHsCTSB or rHsCTSL showed a remarkable reduction of pathological SNCA levels similar to our findings in PD-DA-iPSn. Importantly, positive treatment effects in primary neurons on pathological SNCA conformers could be confirmed by utilizing an ELISA specific for aggregated SNCA. In addition, we show a significant reduction in SNCA-dependent cytotoxicity observed in the primary neurons after administration of both recombinant enzymes. Interestingly, we also observed synaptic reorganization following treatment, countering SNCA-dependent synaptic deficiencies characterized by impaired vesicle recycling, reduced Synapsin-1 expression, and diminished synaptic density [104, 105]. Recovery of synaptic distribution was also evident in recent studies after treatment of synucleinopathy models with recombinant CTSD (rHsCTSD) as well as GCase activators [16, 106], both restoring SNCA pathology while normalizing Synapsin-1 expression and localization. In primary neurons, increased TUBB3 (β3-tubulin signal; a marker of neuronal maturation and cytoskeletal integrity) [107] revealed enhanced neurite branching and complexity, indicating morphological restoration and functional recovery after rHsCTSB and rHsCTSL treatment. These results collectively demonstrate that the therapeutic potential of recombinant CTSB and CTSL extends beyond SNCA reduction, as they also restore lysosomal-autophagic function and neuronal integrity, reinforcing their therapeutic promise. Future studies should elucidate the molecular mechanisms underlying lysosomal functional rescue to fully delineate the treatment’s neuroprotective cascade.

To advance our findings from in vitro studies and establish proof-of-concept for future in-depth in vivo investigations, we tested the therapeutic potential of rHsCTSB and rHsCTSL in a well-characterized in vivo model. For this purpose, we employed intracranial enzyme replacement therapy to bypass the blood-brain barrier - a strategy recently approved for treating pediatric neuronal ceroid lipofuscinosis (CLN2 disease) [108]. Although *Ctsd* knockout mice are not a classical synucleinopathy model, they exhibit profound SNCA aggregation [16]. Intracranial administration of either protease significantly reduced SNCA aggregates, as also previously shown for the aspartic protease CTSD (rHsCTSD) [16]. Our results are complemented by previous work from Di Spiezio et al., who demonstrated that rHsCTSB or rHsCTSL treatment alleviated autophagic dysfunction in *Ctsd* KO mice by reducing accumulated substrates such as Saposin C and LC3-II, although CTSB was less effective than CTSL at degrading aggregated substrates [52]. Follow-up investigations should be conducted in established synucleinopathy in vivo models, such as Thy1-SNCA transgenic mice, to rigorously assess the effects of rHsCTSB and rHsCTSL on disease-relevant phenotypes and behavioral outcomes. In the present study, mechanistic insights were derived from Thy1-SNCA-derived organotypic brain slices and primary neuronal cultures, while in vivo proof-of-concept was obtained in the *Ctsd* KO model, selected for its well-defined treatment paradigm and validated dosing and safety parameters. Future studies should further prioritize combinatorial therapeutic strategies, incorporating CTSB, CTSL, and potentially also CTSD [16]. These long-term, combinatorial treatment approaches are important to comprehensively determine the translational potential of protease-based therapeutics in Parkinson’s disease and related disorders.

In conclusion, our findings establish a critical role for CTSB and CTSL in the proteolysis of SNCA. This effect was accompanied by increased lysosomal/autophagy function, improved neuronal and synaptic organization as well as a reduction in SNCA-dependent neurotoxicity. Hence, our work provides compelling evidence for the therapeutic potential of both cysteine proteases and positions them as promising targets for a much-needed disease-modifying therapy for PD as well as other synucleinopathies.

## Supplementary Information

Below is the link to the electronic supplementary material.

Supplementary Material 1

Supplementary Material 2

## Data Availability

Data is available from the authors for well-grounded reasons.

## Abbreviations

Aa: Amino acid
ACTB: β-actin
SNCA/a-synuclein: Synuclein alpha
CBB: Coomassie brilliant blue
Corr.: Corrected
CTSB: Cathepsin B
CTSD: Cathepsin D
CTSL: Cathepsin L
DA: Dopaminergic
DA-iPSn: Induced pluripotent stem cell-derived dopaminergic neurons
DIV: Days in vitro
ERT: Enzyme replacement therapy
FCS: Fetal calf serum
GCase: β-Glucocerebrosidase
i.c.: Intracranially
IF: Immunofluorescence
iPSC: Induced pluripotent stem cell
KO: Knockout
LAMP2: Lysosomal associated membrane protein 2
neurofil.: Neurofilament
ntg: Non-transgenic
PD: Parkinson disease
proCTSB: Proform of CTSB
proCTSL: Proform of CTSL
rHsCTSB: Recombinant human proCTSB
rHsCTSL: Recombinant human CTSL
sc: Single chain form
hc: Heavy chain form
T-insol: Triton-insoluble
T-sol: Triton-soluble
tg: Transgenic
WT: Wildtype
