# Supplementary material for: Recombinant cathepsins B and L promote α-synuclein clearance and restore lysosomal function in human and murine models with α-synuclein pathology
Source: Mol Neurodegener. 2025 Aug 29;20:95. doi: 10.1186/s13024-025-00886-1 (PMC12398189; doi:10.1186/s13024-025-00886-1)
Supplement: Supplementary file 1 — Supplementary Material 1 [file 13024_2025_886_MOESM1_ESM.docx]

**Supplementary-1 Figures**

**
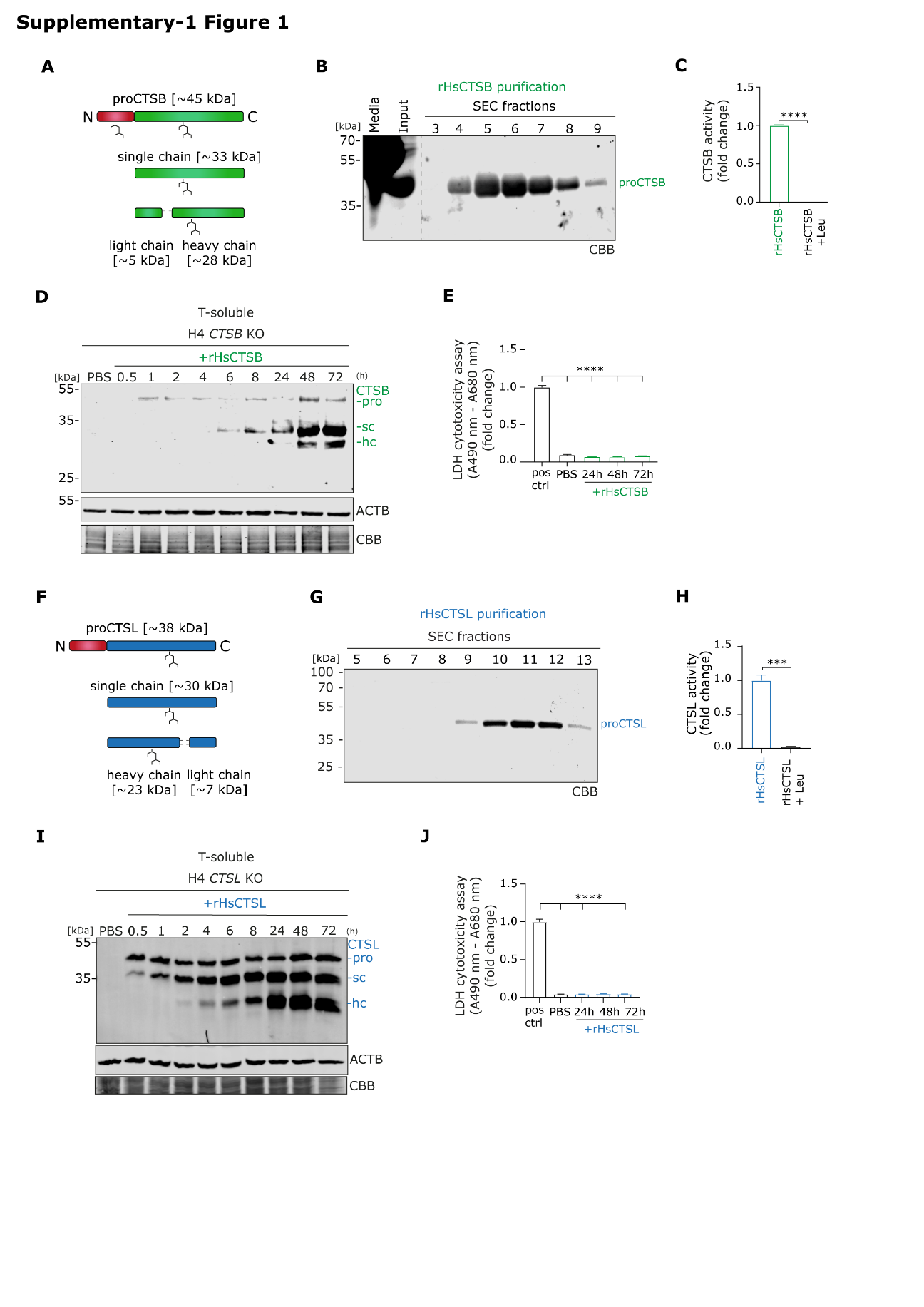
**

**Supplementary-1 Figure 1.** **Production and maturation of recombinant human proCTSB (rHsCTB) and proCTSL (rHsCTSL).**

**(A)** Graphical representation of CTSB maturation: Inactive procathepsin B (proCTSB; ~45 kDa) is processed into an active single chain (sc; ~33 kDa) and subsequently into a double chain consisting of a heavy chain (hc; ~28 kDa) and a light chain (~5 kDa). **(B)** CBB staining of recombinant proCTSB after size exclusion chromatography (SEC). **(C)** CTSB activity assay of SEC fractions and combined rHsCTSB, including Leupeptin (Leu) as a negative control (n = 5 independently purified rHsCTSB). **(D)** Western blot of H4 CTSB KO cells treated with 20 µg/mL rHsCTSB for 0.5 to 72 h, showing proform (~45 kDa), sc (~33 kDa), and hc (~28 kDa) of CTSB. ACTB and CBB were used as loading controls. **(E)** LDH assay of H4 CTSB KO cells treated with 20 µg/mL rHsCTSB for 24, 48, and 72 h. Data were normalized to the positive control and expressed as fold change (n = 3). **(F)** Graphical representation of CTSL maturation: procathepsin L (proCTSL; ~39 kDa) is processed into an active sc form (~30 kDa), which may further cleave into a hc (~23 kDa) and a light chain (~5–7 kDa). **(G)** CBB staining of purified rHsCTSL after SEC. **(H)** CTSL activity assay of SEC fractions and combined rHsCTSL, including Leupeptin (n = 3 independently purified rHsCTSL). **(I)** Western blot of H4 CTSL KO cells treated with 20 µg/mL rHsCTSL for 0.5 to 72 h, showing proform (~38 kDa), sc (~30 kDa), and hc (~23 kDa). ACTB and CBB were used as controls. **(J)** LDH assay of H4 CTSL KO cells treated with rHsCTSL. Data were normalized to LDH positive control and expressed as fold change (n = 3). Data represent mean ± SEM. Statistical analysis: Student’s t-test **(C, H)** or one-way ANOVA with Dunnett’s test **(E, J)**. ****p < 0.0001, ***p < 0.001, *p < 0.05.

**
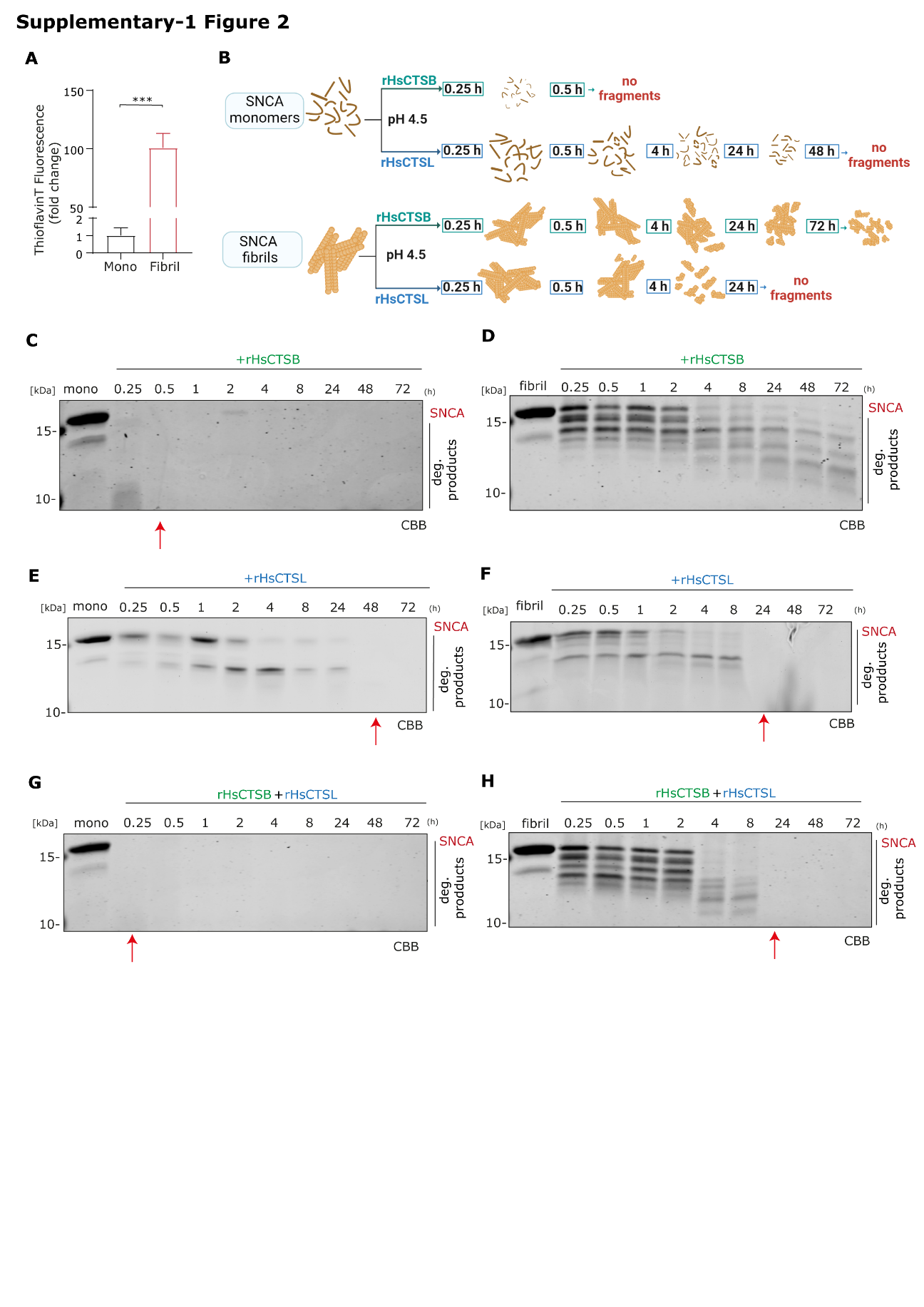
**

**Supplementary-1 Figure 2. *In vitro* digest of SNCA monomer and fibrils by rHsCTSB and rHsCTSL.**

**(A)** Quality control of generated SNCA fibrils was performed using a Thioflavin T assay, comparing the fluorescence intensities of SNCA monomers and fibrils. Data normalized to monomer control and expressed as fold change (n = 3). Data represent mean ± SEM. Statistical analysis: Student’s t-test. ***p < 0.001. *In vitro* degradation assay of monomeric and fibrillar SNCA **(B)** Schematic representation of SNCA monomer and fibril degradation by rHsCTSB and rHsCTSL over time intervals ranging from 0.25 to 72 h at pH 4.5. CBB-stained SDS-PAGE gels illustrate cleavage pattern of monomeric and fibrillar SNCA at pH 4.5 for up to 72 h incubation with recombinant cathepsins. **(C)** Degradation of SNCA monomers by rHsCSB. **(D)** Clearance efficiency of rHsCTSB toward SNCA fibrils. Degradative effect of rHsCTSL on SNCA **(E)** monomers and **(F)** fibrils. Synergistic degradation of SNCA **(G)** monomers and **(H)** fibrils by a combined treatment with rHsCTSB and rHsCTSL.

**
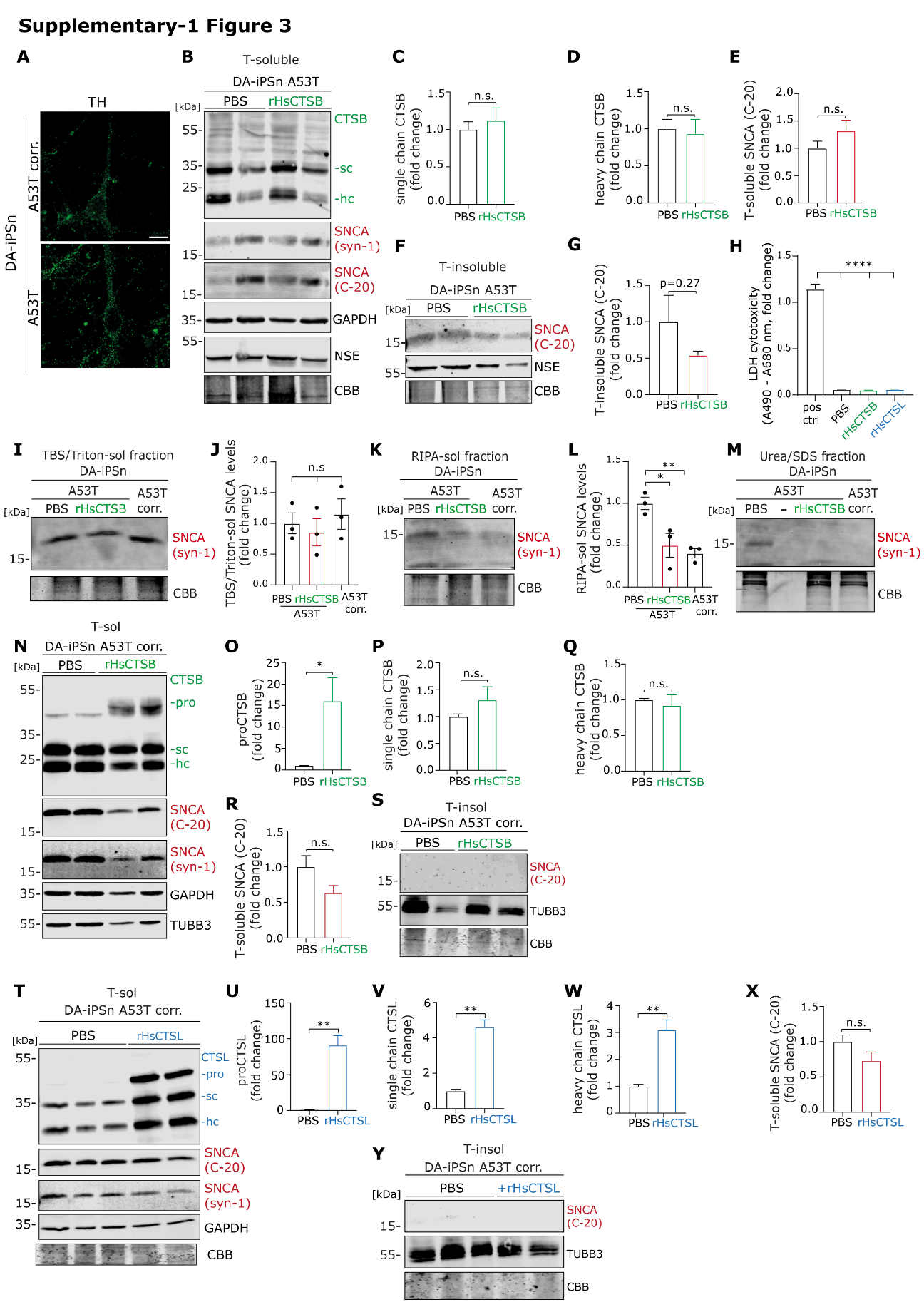
**

**Supplementary Figure 3.** **DA-iPSn from PD patients and isogenic controls treated with rHsCTSB and rHsCTSL.**

**(A)** IF images of DA-iPSn A53T and A53T corr. cells stained with the dopaminergic marker tyrosine hydroxylase (TH; green). Scale bar: 10 µm. **(B)** Western blot of Triton-soluble (T-sol) lysates from DA-iPSn A53T cells treated with PBS or 10 µg/mL rHsCTSB for 17–18 days. SNCA was stained with C-20 and syn-1 antibodies, with GAPDH, NSE, and CBB used as loading controls. Quantification of **(C)** sc, **(D)** hc of CTSB, and **(E)** soluble SNCA (C-20) was normalized to GAPDH (n = 5). **(F)** Western blot of Triton-insoluble/SDS-soluble (T-insol) lysates from DA-iPSn A53T cells treated with PBS or rHsCTSB, with **(G)** quantification of insoluble SNCA (C-20) normalized to NSE (n = 3). **(H)** LDH assay of DA-iPSn A53T cells treated with PBS, rHsCTSB, or rHsCTSL (21–25 days), normalized to positive control (n = 3). **(I)** Representative Western blot of TBS/Triton soluble fraction of lysates from DA-iPSn, A53T cells treated with PBS or CTSB and the isogenic corrected line. SNCA was stained using with syn-1 antibody with CBB used as a loading control. **(J)** Quantification of TBS/Triton-soluble SNCA (syn-1) levels normalized to CBB and expressed as fold-change to A53T treated with PBS (n = 3). **(K)** Representative Western blot of RIPA soluble fraction of lysates from DA-iPSn, with SNCA stained by the syn-1 antibody and CBB used as loading control. **(L)** Quantification of RIPA-soluble SNCA (syn-1) levels normalized to CBB and expressed as fold-change to PBS treated A53T line (n = 3). **(M)** Representative Western blot of Urea/SDS fraction of lysates from DA-iPSn, stained with syn-1 for SNCA and CBB used as loading control. **(N)** Western blot of T-sol lysates from DA-iPSn A53T corr. cells treated with PBS or 10 µg/mL rHsCTSB for 21–25 days, stained for CTSB, C-20, and syn-1. GAPDH, TUBB3, and CBB were used as controls. Quantification of **(O)** proform, **(P)** sc, **(Q)** hc of CTSB, and **(R)** soluble SNCA was normalized to GAPDH (n = 3). **(S)** Western blot of T-insol lysates from DA-iPSn A53T corr. cells treated with PBS or rHsCTSB. **(T)** Western blot of T-sol lysates from DA-iPSn A53T corr. cells treated with PBS or 10 µg/mL rHsCTSL, with GAPDH and CBB as controls. Quantification of **(U)** proform, **(V)** sc, **(W)** hc of CTSL, and **(X)** soluble SNCA normalized to GAPDH (n = 3). **(X)** Western blot of T-insol lysates from DA-iPSn A53T corr. treated with PBS or rHsCTSL. Data represent mean ± SEM. Statistical analyses: Student’s t-test relative **(C, D, E, G, O, P, Q, U, V, W, X)** or one-way ANOVA with Dunnett’s test **(H, J, L)**. Statistical differences are shown toward PBS treated A53T mutant cells. ****p < 0.0001, **p < 0.01, *p < 0.05; n.s., not significant.

**
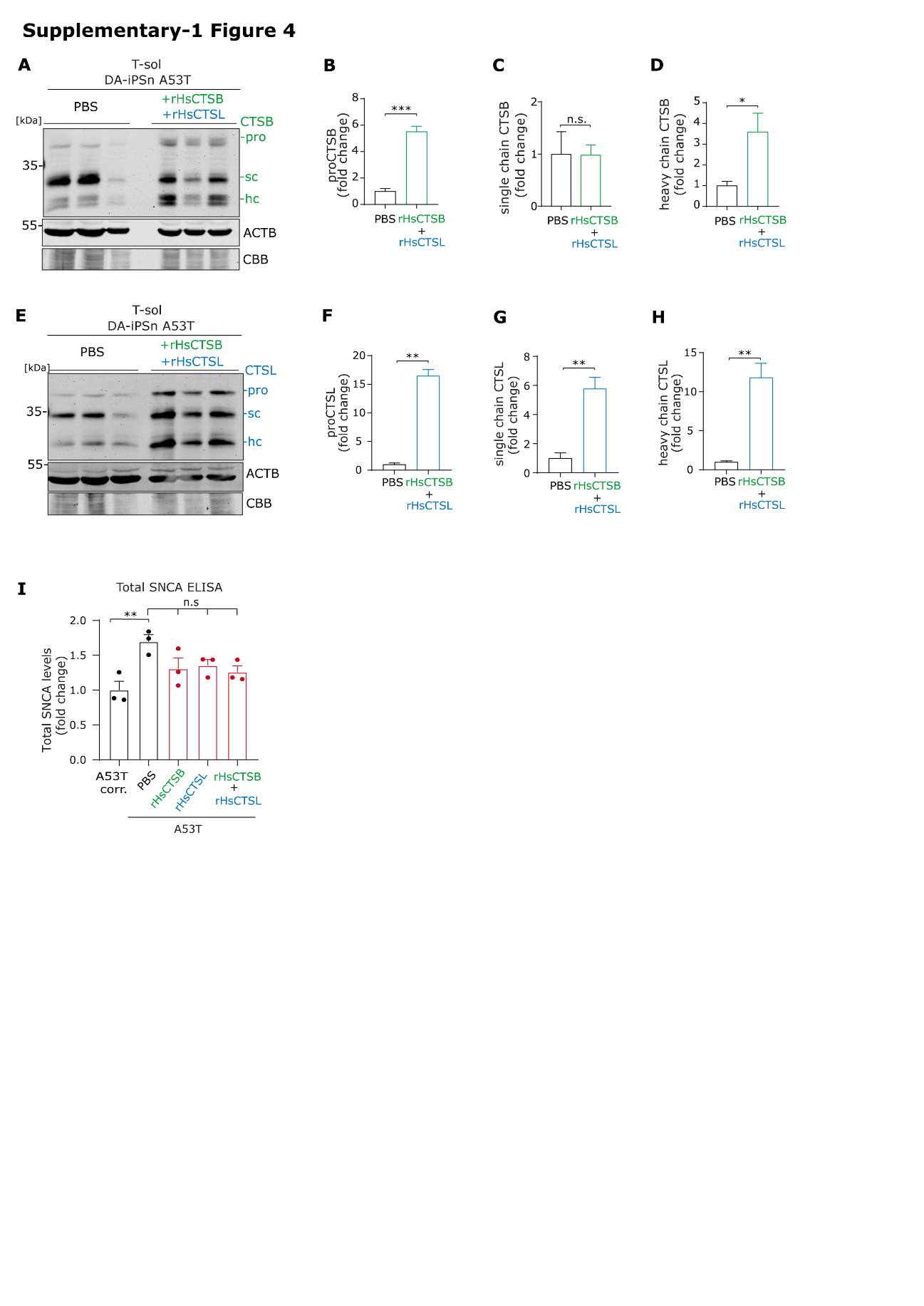
**

**Supplementary-1 Figure 4. Combined treatment of rHsCTSB and rHsCTSL in DA-iPSn A53T cells.**

**(A)** Representative Western blot of Triton-soluble (T-sol) lysates of DA-iPSn A53T treated with PBS or a combination of 10 µg/mL rHsCTSB and rHsCTSL for 21-25 days and stained for CTSB. ACTB and CBB were used as loading controls. Quantification of **(B)** the proform, **(C)** the sc and **(D)** hc of CTSB was normalized to ACTB and expressed as fold change (n = 3). **(E)** Representative Western blot of T-sol lysates of DA-iPSn cells treated with PBS or a combination of rHsCTSB and rHsCTSL, stained for CTSL with beta-actin (ACTB) and CBB were used as loading controls. Quantification of **(F)** the proform, **(G)** the sc and **(H)** hc of CTSL was normalized to ACTB and expressed as fold change (n = 3). **(I)** Analysis of total SNCA ELISA showing levels of total soluble SNCA within the DA-iPSn A53T with PBS or 10 µg/mL rHsCTSB, rHsCTSL or the combination of rHsCTSB and rHCTSL treatment and expressed as fold change to A53T corrected line (n = 3). All data represent mean ± SEM. Statistical analyses were performed by using a two-tailed unpaired Student’s t-test **(B, C, D, F, G, H)** or one-way ANOVA with Dunnett’s test **(I)**. Statistical differences are shown toward PBS treated A53T mutant cells. ****p < 0.0001, ***p < 0.001, **p < 0.01, *p < 0.05; n.s., not significant.

**
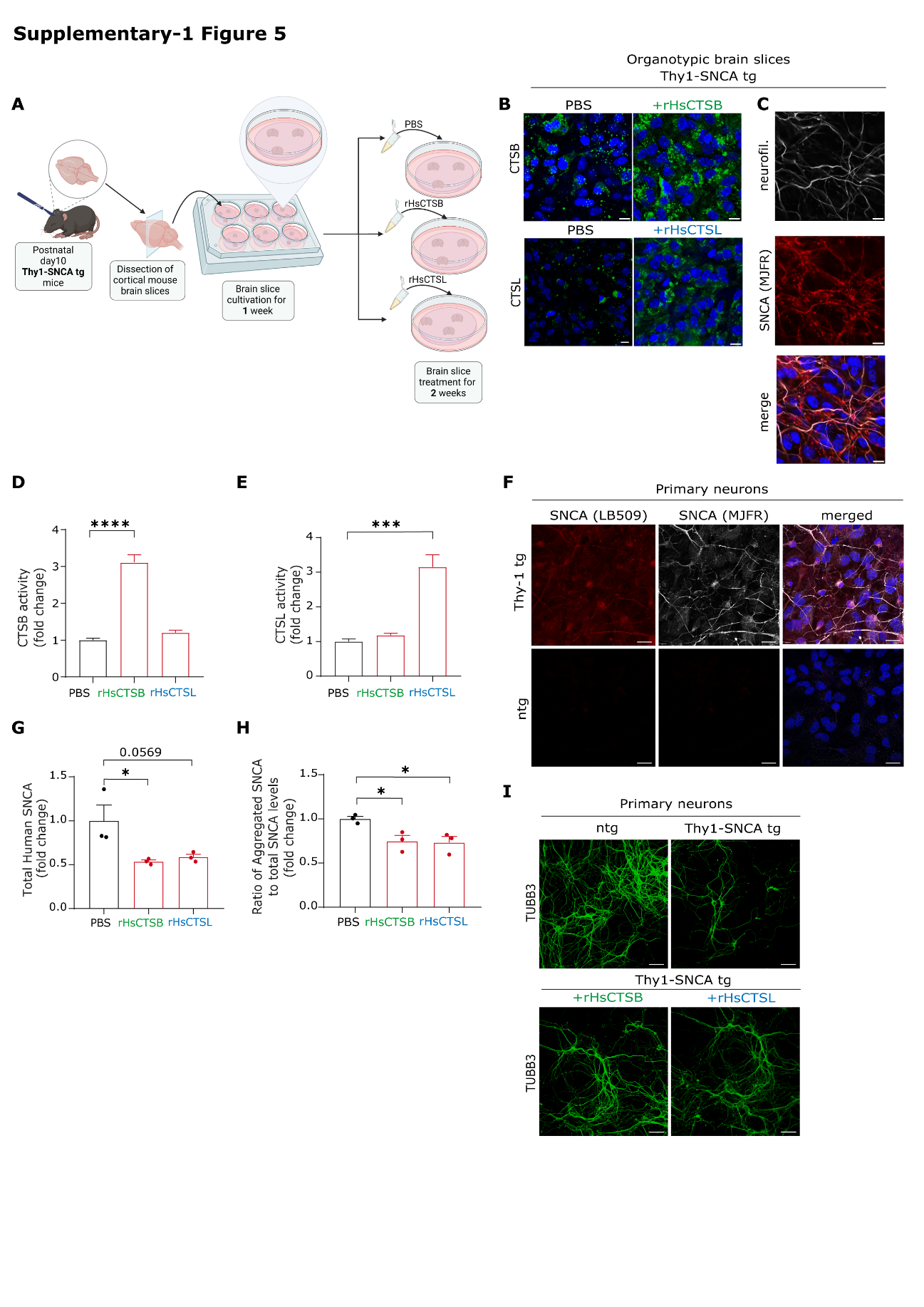
**

**Supplementary-1 Figure 5.** **Effects of CTSB or CTSL treatment of organotypic brain slices and primary neurons derived from transgenic Thy1-SNCA mice.** **(A)** Schematic illustration of cortical brain slice generation and cultivation derived from postnatal day 10 transgenic (tg) Thy1-SNCA mice. **(B)** Representative IF stainings of tg Thy 1-SNCA mice brain slices treated with 20 µg/mL rHsCTSB or 20 µg/mL rHsCTSL and the same volume of PBS to demonstrate uptake of the lysosomal enzyme. Images show distribution of CTSB and CTSL within the tissue (in green). DAPI staining in blue. Scale bar: 10 µm. **(C)** IF pictures of neurons using the neuronal marker neurofilament (neurofil., grey) and the pathology-associated SNCA antibody (MJFR-14-6-4-2; red). DAPI staining in blue. Scale bar: 10 µm. Lysate activity assay for **(D)** CTSB and **(E)** CTSL for primary neurons treated with rHsCTSB and rHsCTSL to validate the uptake of the enzymes. Data represented as fold change to PBS treated neurons from Thy1-SNCA tg mice (n = 3). **(F)** Representative IF images of primary neurons from Thy-1 SNCA tg and ntg mice stained for pathology-associated LB509 (red) and MJFR-14-6-4-2 (grey) antibodies. DAPI staining in blue. Scale bar: 20 µm. **(G)** Analysis of total SNCA ELISA showing levels of total soluble human- SNCA within the primary neurons from Thy1-tg treated with rHsCTSB and rHsCTSL and expressed as fold change to PBS treated control (n=3). **(H)** Ratio of Aggregate SNCA to total SNCA in primary neurons as determined from the quantification of the aggregate ELISA (Figure 5G) and total human-SNCA (Figure S5G) and expressed as fold-change to PBS treated control (n = 3). **(I)** Representative IF pictures of primary neurons from ntg mice as well as Thy1-SNCA tg mice treated with PBS, rHsCTSB or rHsCTSL, stained for TUBB3 (green) as a neuronal marker. Scale bar: 20 µm. All data represent mean ± SEM. Statistical analyses were performed by using a one-way ANOVA with Dunnett’s test **(D, E, G, H)**. Statistical differences are shown toward PBS treated primary neurons derived transgenic Thy1-SNCA mice. ****p < 0.0001, ***p < 0.001, **p < 0.01, *p < 0.05; n.s., not significant.


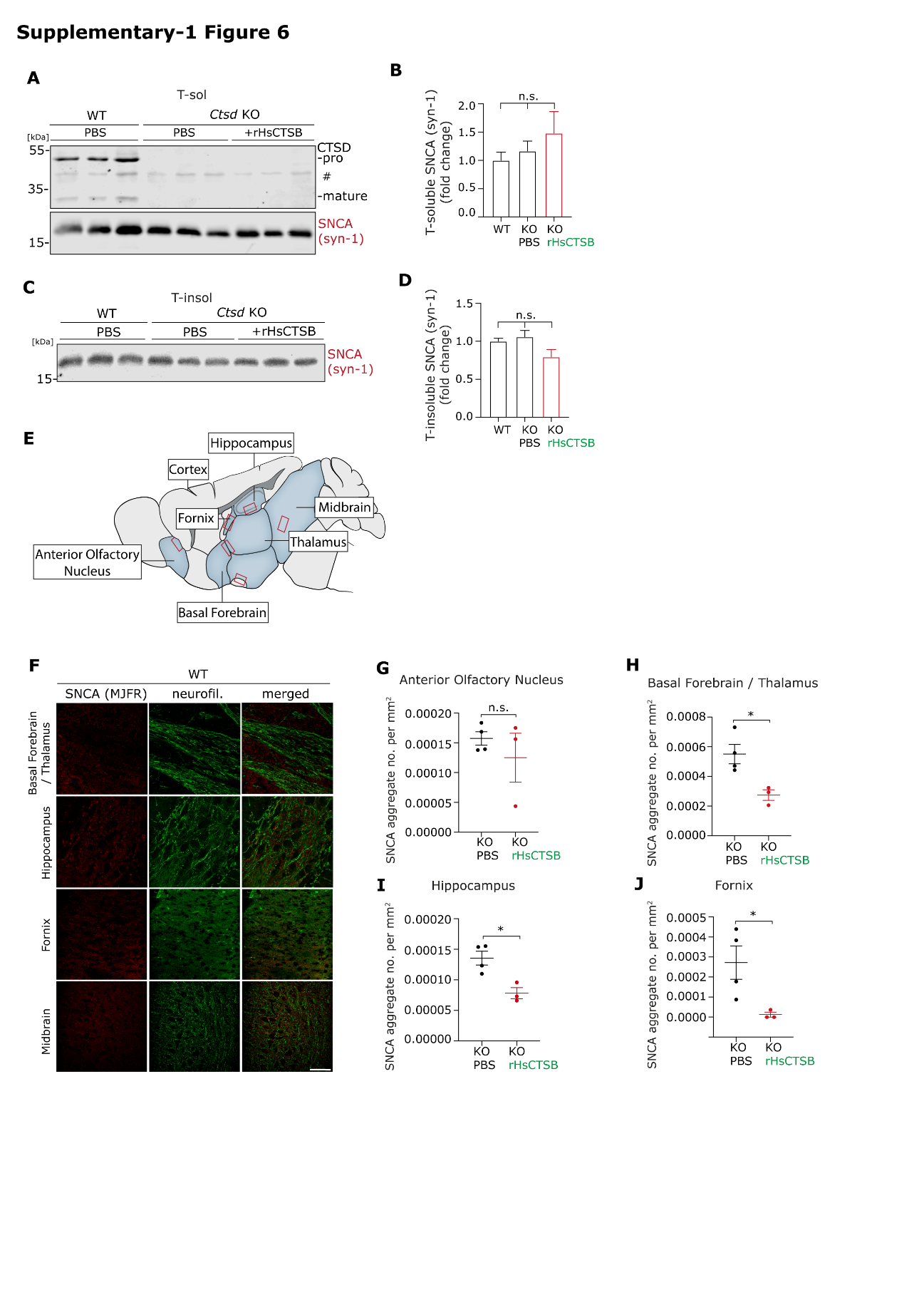


**Supplementary-1 Figure 6 – Analysis of cathepsins and SNCA in *Ctsd* knockout mice.**

**(A)** Corresponding staining of CTSD and SNCA syn-1 from Western blot shown in Figure 6A. An unspecific band detected by CTSD antibody was present in all samples and labeled with the symbol ‘#’. **(B)** Quantification of soluble SNCA signal by syn-1 antibody (n = 3 mice per group). **(C)** Corresponding staining of SNCA syn-1 from Western blot shown in Figure 4E. **(D)** Quantification of Triton-insoluble/SDS-soluble SNCA signal by syn-1 antibody (n = 3 mice per group). **(E)** Schematic representation of the mouse brain in a sagittal cut. The region marked by the red square shows where the confocal images were taken. **(F)** Representative confocal microscopy pictures of WT mouse brain treated with PBS. Pathology-associated SNCA was detected by conformation-specific antibody MJFR-14-6-4-2 (red) and costained with neurofilament (green). Scale bar: 40x. Quantification of the number of SNCA aggregates per mm^2^ revealed in the **(G)** anterior olfactory nucleus, **(H)** basal forebrain/thalamus, **(I)** hippocampus and **(J)** fornix. All data represent mean ± SEM. Statistical analyses were performed by using a one-way ANOVA together with Tukey’s multiple comparison test **(B, D)** or a two-tailed unpaired Student’s t-test **(F–I)**. Statistical differences are shown toward WT **(B, D)**. *p < 0.05; n.s., not significant.


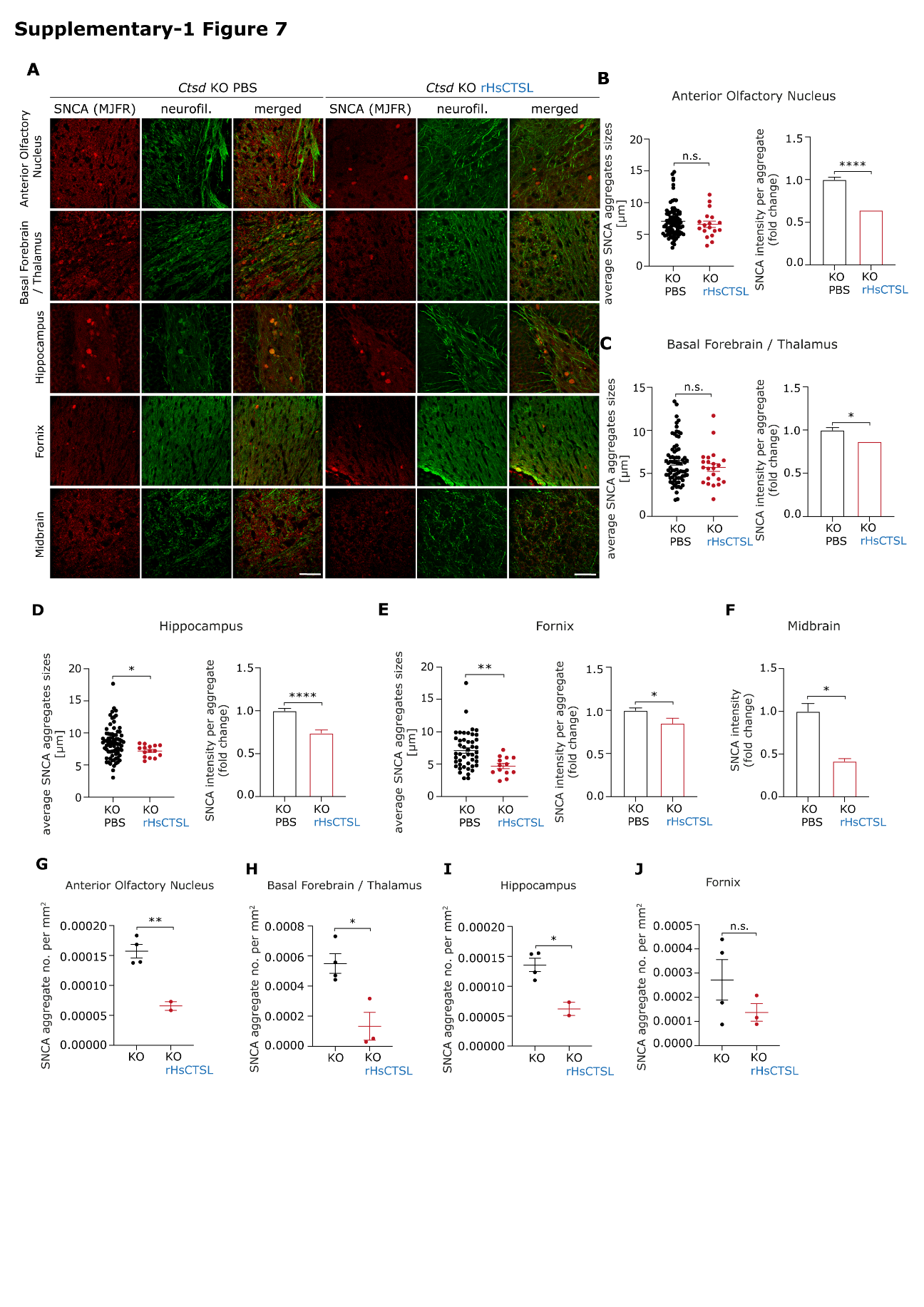
**Supplementary-1 Figure 7.** **Proof-of-concept *in-vivo* study:** **intracranial injection of rHsCTSL in *Ctsd* KO mice brain.**

**(A)** Representative images of *Ctsd* KO brains intracranially injected with 10 µL PBS or 100 µg rHsCTSL in 10 µL PBS (KO PBS) and *Ctsd* KO +rHsCTSL. Pathology-associated SNCA was stained with the conformation-specific antibody MJFR-14-6-4-2 (red) and neurons were stained with neuronal marker neurofilament (neurofil., green). Scale bar: 50 µm. Analyses of average SNCA aggregates size (µm) and SNCA intensity signal per aggregate in the **(B)** anterior olfactory nucleus, **(C)** basal forebrain/thalamus, **(D)** hippocampus, **(E)** fornix, and **(F)** midbrain. Dots represent SNCA aggregates found per image in the respective brain area (n = 2-3 mice per group). Quantification of the number of SNCA aggregates per mm^2^ revealed in the **(G)** anterior olfactory nucleus, **(H)** basal forebrain/thalamus, **(I)** hippocampus and **(J)** fornix. All data represent mean ± SEM. Statistical analyses were performed by using a two-tailed unpaired Student’s t-test **(B-J)** in which differences are shown toward PBS treatment. p < 0.0001, **p < 0.01, *p < 0.05; n.s., not significant.
