# Supplementary material for: Recombinant cathepsins B and L promote α-synuclein clearance and restore lysosomal function in human and murine models with α-synuclein pathology
Source: Mol Neurodegener. 2025 Aug 29;20:95. doi: 10.1186/s13024-025-00886-1 (PMC12398189; doi:10.1186/s13024-025-00886-1)
Supplement: Supplementary file 2 — Supplementary Material 2 [file 13024_2025_886_MOESM2_ESM.docx]

**Supplementary file – 2**


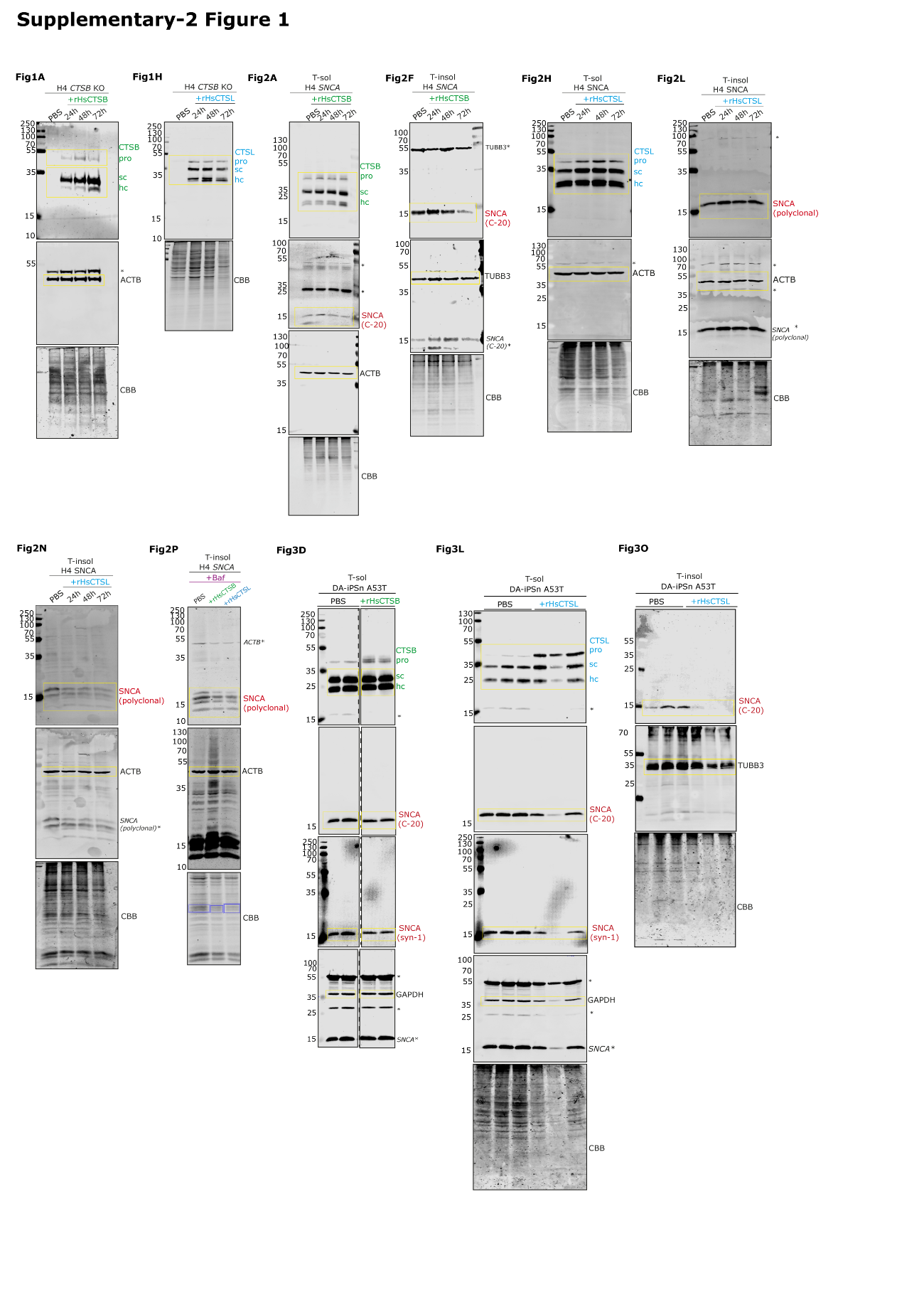


**Supplementary-2 Figure 1. Full-length blots corresponding to Figures 1-3.**

MW shown in kilodaltons (kDa). Asterisks indicate irrelevant bands (observed by secondary antibody alone or residual bands from a previous staining). Yellow boxes indicate the specific protein stained. See corresponding figures in the main manuscript for full explanations.


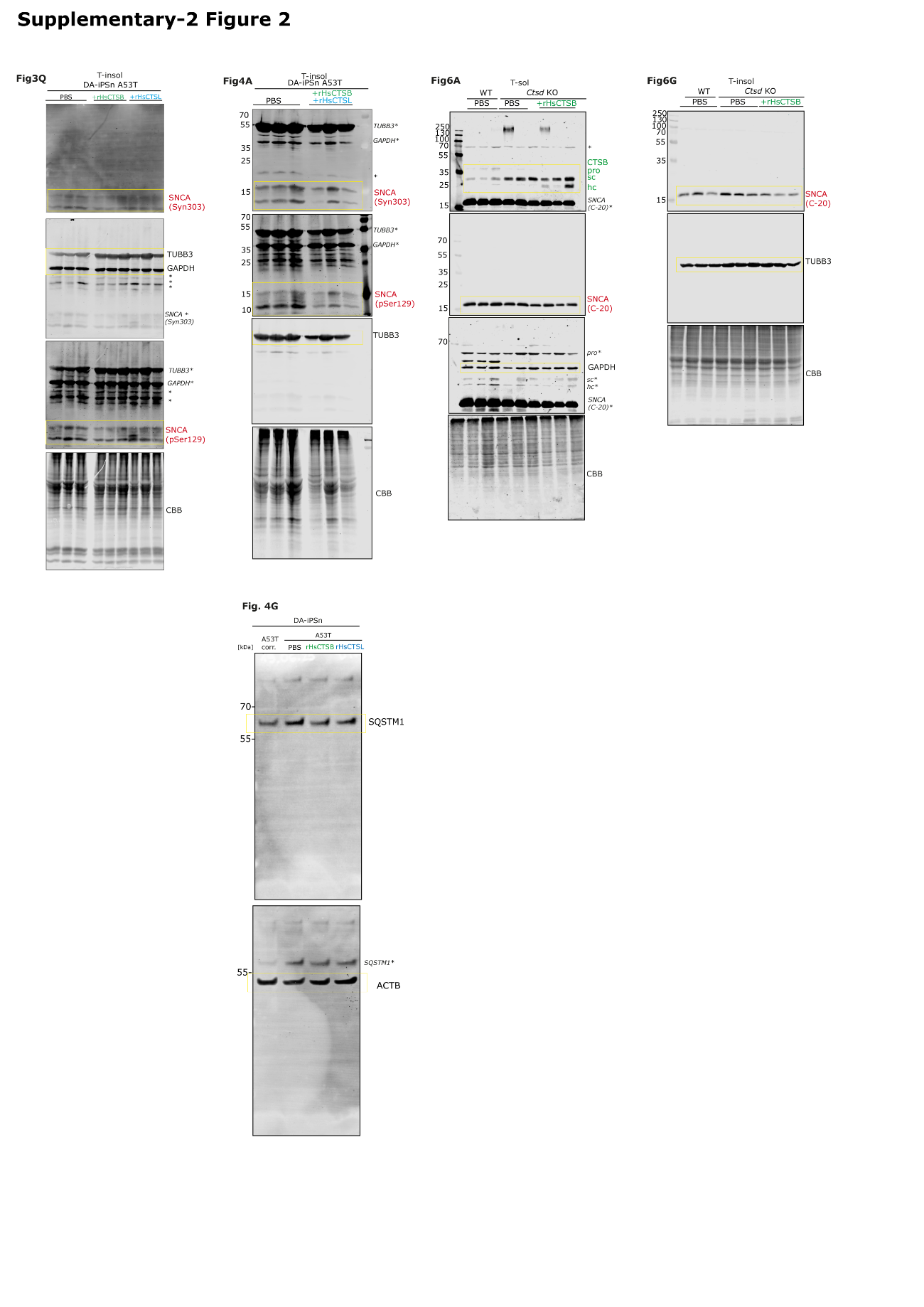


**Supplementary-2 Figure 2. Full-length blots corresponding to Figures 3-6.**

MW shown in kilodaltons (kDa). Asterisks indicate irrelevant bands (observed by secondary antibody alone or residual bands from a previous staining). Yellow boxes indicate the specific protein stained. See corresponding figures in the main manuscript and Supplementary-2 Figure 4 for full explanations.


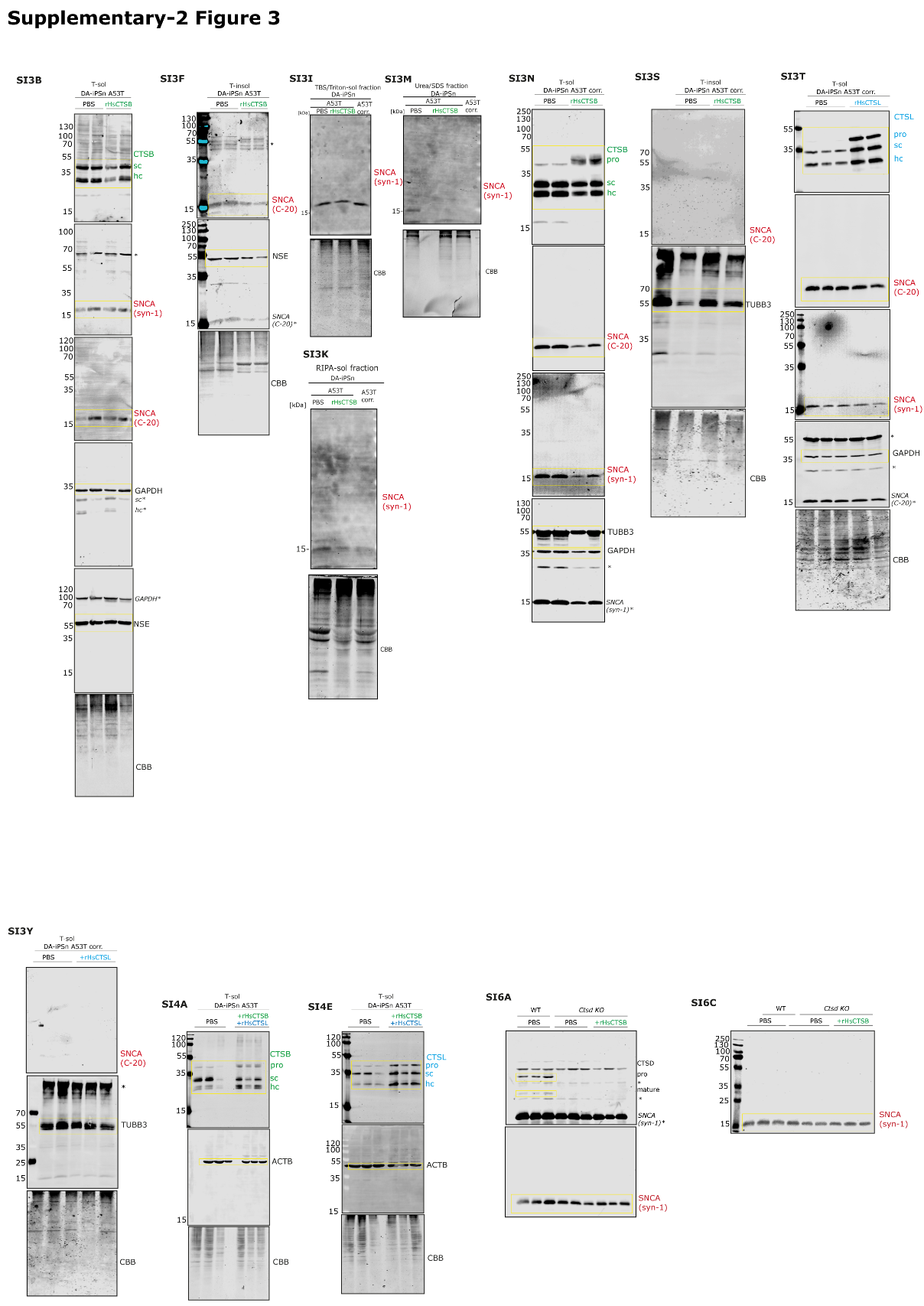


**Supplemental file-2 Figure 3. Full-length blots corresponding to Figures shown in Supplementary file-1 Figures S1-S7.**

MW shown in kilodaltons (kDa). Asterisks indicate irrelevant bands (observed by secondary antibody alone or residual bands from a previous staining). Yellow boxes indicate the specific protein stained. See corresponding figures in the main manuscript for full explanations.

**
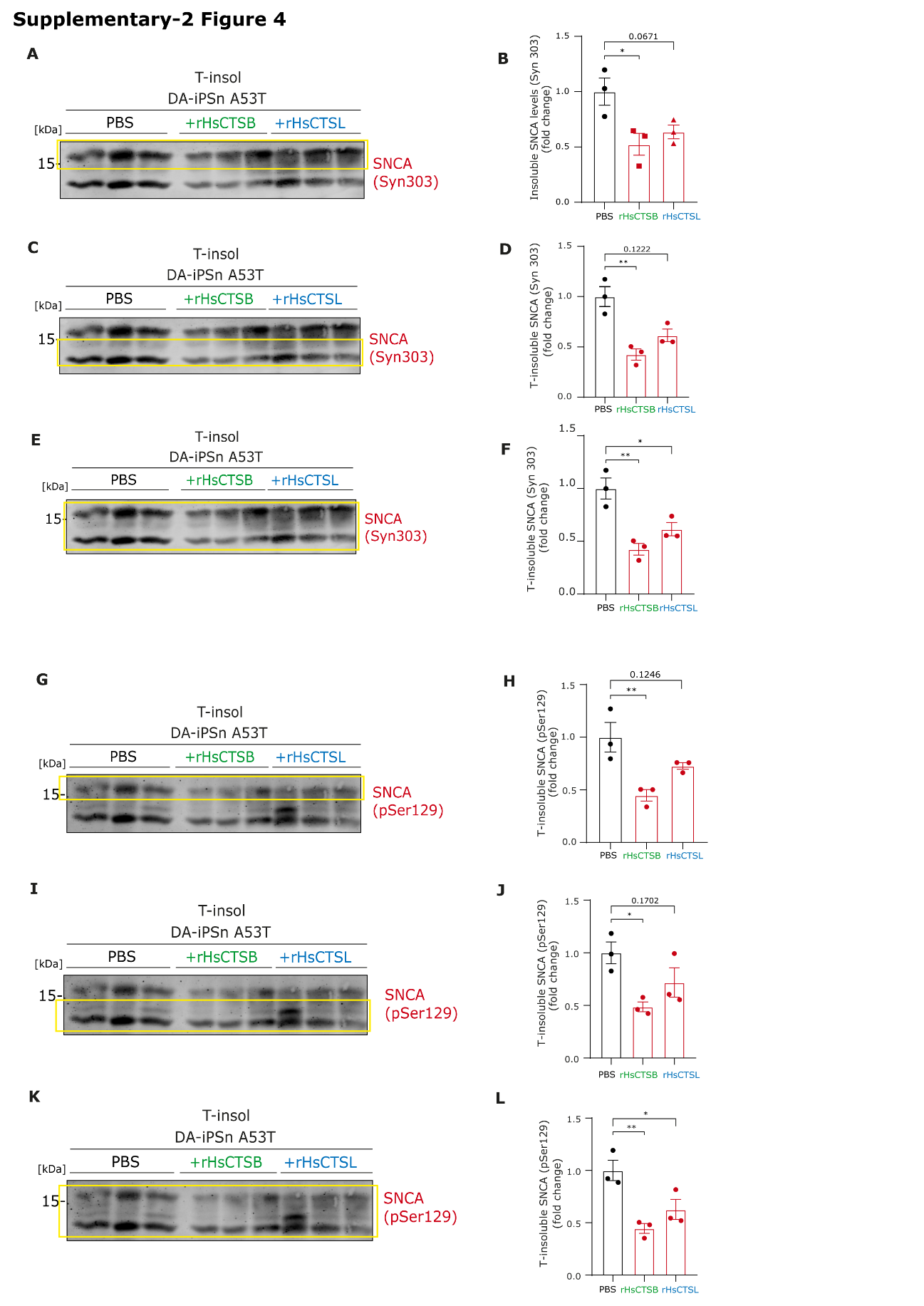
**

**Supplemental file-2 Figure 4 Effects of rHsCTSB and rHsCTSL treatment on different species of SNCA from Figure 3Q as a representative example.**

Western blots corresponding to Figure 3Q shown in the main manuscript, where Triton-insoluble (T-insol) protein fraction from A53T DA-iPSn were probed with Syn303 and pSer129 antibodies for SNCA, with 40µg protein loaded per condition, to study the effects of treatment with rHsCTSB and rHsCTSL.

Lower molecular weight species of SNCA were detected and bands corresponding to full-length SNCA (~15 kDa) as well as lower molecular weight (LMW) forms were analysed.

**(A)** and **(G)** shows the representative pictures of the blots where full-length SNCA was analysed after staining for Syn303 and pSer129 respectively, with **(B)** and **(H)** showing the corresponding quantification of the effect of treatment on the full-length SNCA, as shown in the analyses in the main-manuscript Figure 3S and 3T. **(C)** and **(I)** depicts representative pictures for LMW species of SNCA stained by Syn303 and pSer129 respectively, with **(D)** and **(J)** showing the corresponding quantification of the effects of treatment on LMW forms of SNCA. **(E)** and **(K)** correspond to representative pictures showing where all bands immunoreactive to SNCA using Syn303 and pSer129 respectively were analysed, with **(F)** and **(L)** showing the corresponding quantification of total SNCA species after treatment. Yellow boxes around the blots reflect the region being analysed. In all cases, treatment with rHsCTSB and rHsCTSL led to the reduction in SNCA. All data represent mean ± SEM, and expressed as fold change to PBS treated A53T DA-iPSn and normalized to CBB as loading control. Statistical analyses were performed by using one-way ANOVA with Dunnett’s test in which differences are shown toward PBS treatment. p < 0.0001, **p < 0.01, *p < 0.05; n.s., not significant.
